# Decoding how RNase E membrane targeting controls RNA degradosome activity in *Pseudomonas aeruginosa*

**DOI:** 10.1016/j.isci.2026.117717

**Published:** 2026-10-01

**Authors:** Sandra Amandine Marie Geslain, George Edward Allen, Johan Geiser, Peter Redder, Martina Valentini

**Affiliations:** 1 Department of Microbiology and Molecular Medicine, Faculty of Medicine, University of Geneva, 1211 Geneva, Switzerland; 2 Laboratoire de Microbiologie et Génétique Moléculaires (LMGM), Centre de Biologie Intégrative (CBI), Université Toulouse III, CNRS, 31400 Toulouse, France; 3 Institute of Genetics and Genomics in Geneva (iGE3), University of Geneva, 1211 Geneva, Switzerland

**Keywords:** RNA degradosome, RNA localization, gram-negative bacteria, *Pseudomonas aeruginosa*, RNase E, amphipathic helix

## Abstract

In many Proteobacterial species, transcription and RNA degradation are physically separated through segregation of the main ribonucleolytic machinery—the RNA degradosome—into RNase E-driven membrane-anchored assemblies that form discrete foci. Despite widespread conservation of the amphipathic membrane-targeting sequence (MTS) in RNase E, its function and biological importance remain poorly understood. Here, we have investigated how the MTS contributes to bacterial virulence or stress response in *Pseudomonas aeruginosa*. We show that membrane anchoring is dispensable for formation of RNA degradosome foci, but required for their proper morphology, dynamics, and localization, whereas the MTS sequence modulates foci behavior. Transcriptomic and mRNA spatial organization analyses further reveal specific stabilization of localized transcripts encoding membrane proteins in the MTS mutant, together with abnormal operon processing. Altogether, our study reveals the elegant MTS-mediated control of RNA degradosome spatial organization and target selection, shaping the transcriptome and bacterial stress responses.

## Highlights

• RNase E membrane-targeting sequence (MTS) controls RNA degradosome localization
• MTS is dispensable for RNA degradosome foci formation but controls their dynamics
• MTS loss alters bacterial environmental adaptation and virulence
• MTS loss stabilizes transmembrane protein transcripts and alters operon processing

## Graphical abstract

**Figure:**
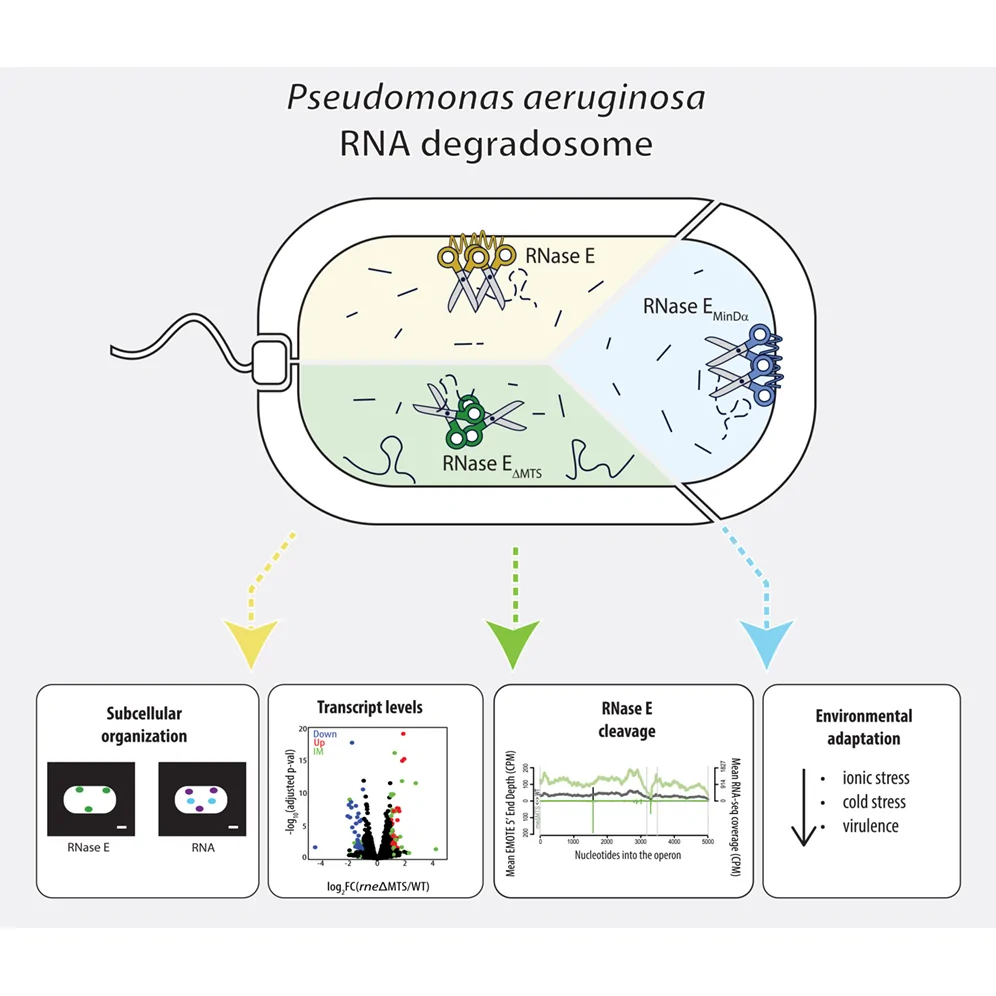

## Teaser

Molecular microbiology; Transcriptomics

## Introduction

Cells in all domains of life encompass spatial organization that supports regulation and other key functions. Initially discovered in eukaryotic cells, intricate cytosolic organization has also been observed in bacterial cells.^1^ Notably, despite lacking membrane-bound organelles, bacteria are able to separate core cellular processes such as transcription, translation, or degradation by excluding or localizing specific machinery to the cell pole, the midcell region, or the membrane.^1^ Such organization is largely driven by the nucleocytoplasmic ratio (NC ratio), a feature that shapes the biophysical properties of the cytoplasm.^2^ Bacteria with a high NC ratio, such as *Caulobacter crescentus*, exhibit a nucleoid that occupies most of the cytosol: ribosomes are mixed with the DNA mesh and diffusion rates are greatly decreased, causing most mRNAs to remain near their transcription site throughout most of their lifetime. On the other hand, in bacteria with a low NC ratio such as *Escherichia coli*, the nucleoid is restricted to the middle of the cell, and it excludes ribosomes, which are thus found enriched near the cell poles or the membrane.^1^^,^^2^ In these bacteria, mRNAs are not enriched near their genomic loci and are instead found in various cellular locations throughout the entire cell, with most mRNAs coding for inner membrane proteins localizing near the membrane.^3^^,^^4^

The RNA degradosome, a key multiprotein complex involved in the processing of mRNAs, maturation of rRNA or tRNA, and in RNA decay, also exhibits a distinct subcellular localization.^5^^,^^6^^,^^7^ In *C. crescentus*, and in other species with a high NC ratio, the RNA degradosome assembles into large cytoplasmic foci termed bacterial ribonucleoprotein bodies (BR-bodies), organized by its core component RNase E.^2^^,^^8^^,^^9^ BR-body assembly is RNA-dependent and occurs through RNase E-mediated liquid-liquid phase separation.^8^ In most Gammaproteobacteria, the RNA degradosome also localizes as foci, but RNase E additionally harbors a conserved amphipathic helix of approximately 15 residues, called the membrane targeting sequence (MTS). In *E. coli*, the MTS positions the foci near the membrane,^10^^,^^11^^,^^12^ and an MTS-deleted *E. coli* RNase E exhibits a diffuse and cytoplasmic localization, indicating that the MTS is required for both membrane binding and foci assembly.^11^^,^^13^ In addition, a recent study has shown that an *E. coli* RNase E mutant lacking the MTS exhibits a slower growth rate and accumulates high levels of 20S and 40S aberrant ribosomal particles, which the authors attribute to a premature cleavage of rRNA precursors resulting in accumulation of abnormally folded rRNA particles.^14^ These results are consistent with a model in which separation of the RNA degradosome from the nucleoid avoids futile degradation of ribosome assembly intermediates, a reasoning that likely also applies to nascent mRNAs.^5^^,^^7^^,^^14^

Several studies have shown that the interplay between mRNA localization and RNA degradosome spatial organization influences mRNA half-life. Moffitt et al.^3^ demonstrated that, in *E. coli*, mRNAs encoding inner membrane proteins exhibit shorter half-lives than other transcripts, a difference which is abolished in a strain expressing an RNase E variant lacking the MTS.^3^ Notably, the MTS-deleted strain also displays a global increase in mRNA half-life, a phenotype independently reported by Hadjeras et al.,^15^ consistent with earlier observations that delocalization of RNase E from the membrane can impair its catalytic activity.^15^^,^^16^ Further supporting the importance of proper RNA degradosome localization, a recent study showed that loss of MinD, which transiently interacts with RNase E via the MTS, leads to RNase E accumulation at the cell poles, resulting in disrupted polar mRNA localization and reduced half-lives of polar transcripts.^17^

Despite the growing evidence about the need to compartmentalize the RNA degradosome, it remains largely unknown how this separation contributes to bacterial fitness and stress adaptation. To our knowledge, the phenotypic effect of RNA degradosome membrane delocalization reported to date is a mild growth defect in rich medium.^14^ In addition, the MTS sequence is known to be rather conserved in all Gammaproteobacteria, but its recently described regulatory interaction with MinD in *E. coli* raises an interesting question: is the MTS involved in additional roles beyond membrane anchoring?

To bridge this gap, we studied membrane anchoring of the RNA degradosome in the WHO high-priority pathogen *Pseudomonas aeruginosa*, a bacterium especially suited to RNA metabolism studies due to its adaptability and intricate regulatory networks.^18^ By comparing a mutant lacking the RNase E MTS with a chimeric RNase E mutant bearing a heterologous amphipathic helix, we have assessed the interchangeability and *in vivo* importance of the RNase E MTS. Overall, our results show that MTS-mediated anchoring is dispensable for assembly of RNA degradosome foci but is required for proper foci dynamics and subcellular localization. Importantly, deletion of the MTS impairs adaptation to specific stresses and virulence in a *Galleria mellonella* model. Furthermore, we uncover a previously unrecognized role for the MTS in RNase E operon-specific mRNA processing, affecting a subset of transcripts encoding inner membrane proteins. Overall, our work establishes a novel role for the RNase E MTS in mRNA target selection and in normal adaptive stress response, likely sustained by the elegant process of MTS-mediated RNA degradosome clustering and dissolution.

## Results

### RNase E MTS deletion redistributes RNA degradosome foci in *P. aeruginosa* and is complemented by MinDα

In Beta and Gammaproteobacteria, most RNase E homologs harbor a membrane-targeting sequence (MTS) defined by a conserved amphipathicity.^5^^,^^10^^,^^19^ We previously confirmed the presence of an MTS within *P. aeruginosa* RNase E (hereafter simply called RNase E) and showed that the protein forms discrete foci at the membrane.^20^ To investigate the subcellular localization of RNase E upon loss of the MTS, we constructed a strain expressing RNase E^ΔMTS^-msfGFP from the native chromosomal locus. Visualization of the signal revealed that the RNase E^ΔMTS^-msfGFP is still capable of assembling foci (Figure 1A); however, these foci have statistically significantly higher fluorescence intensity values and rounder outlines than the ones formed by the full-length RNase E (Figure 1B). Additionally, the average subcellular localization of RNase E^ΔMTS^-msfGFP is shifted compared to the native protein: although foci still tend to be enriched near the cell periphery, we observe a striking increase in foci density near the core region of the cells and a significantly higher frequency of foci located at the cell poles (12.4% vs. 5.7%) and midcell (10% vs. 7.3%) compared to foci formed by the full-length RNase E (Figure 1C). To verify that the foci observed in the RNase E^ΔMTS^-msfGFP were RNA-dependent assemblies and not caused by protein aggregation or misfolding, we treated the cells with rifampicin to deplete cellular RNA due to transcription inhibition. Visualization of the cells after 30 min showed complete dissolution of RNase E^ΔMTS^-msfGFP foci as observed with the full-length RNase E (Figure 1D). Additionally, we observed a completely homogeneous subcellular localization of rifampicin-treated RNase E^ΔMTS^-msfGFP, in contrast to the membrane-enriched peripheral localization of RNase E full-length (Figure 1D), which further confirms that deletion of the MTS delocalizes RNase E, and thereby the RNA degradosome, from the membrane.

**Figure 1:**
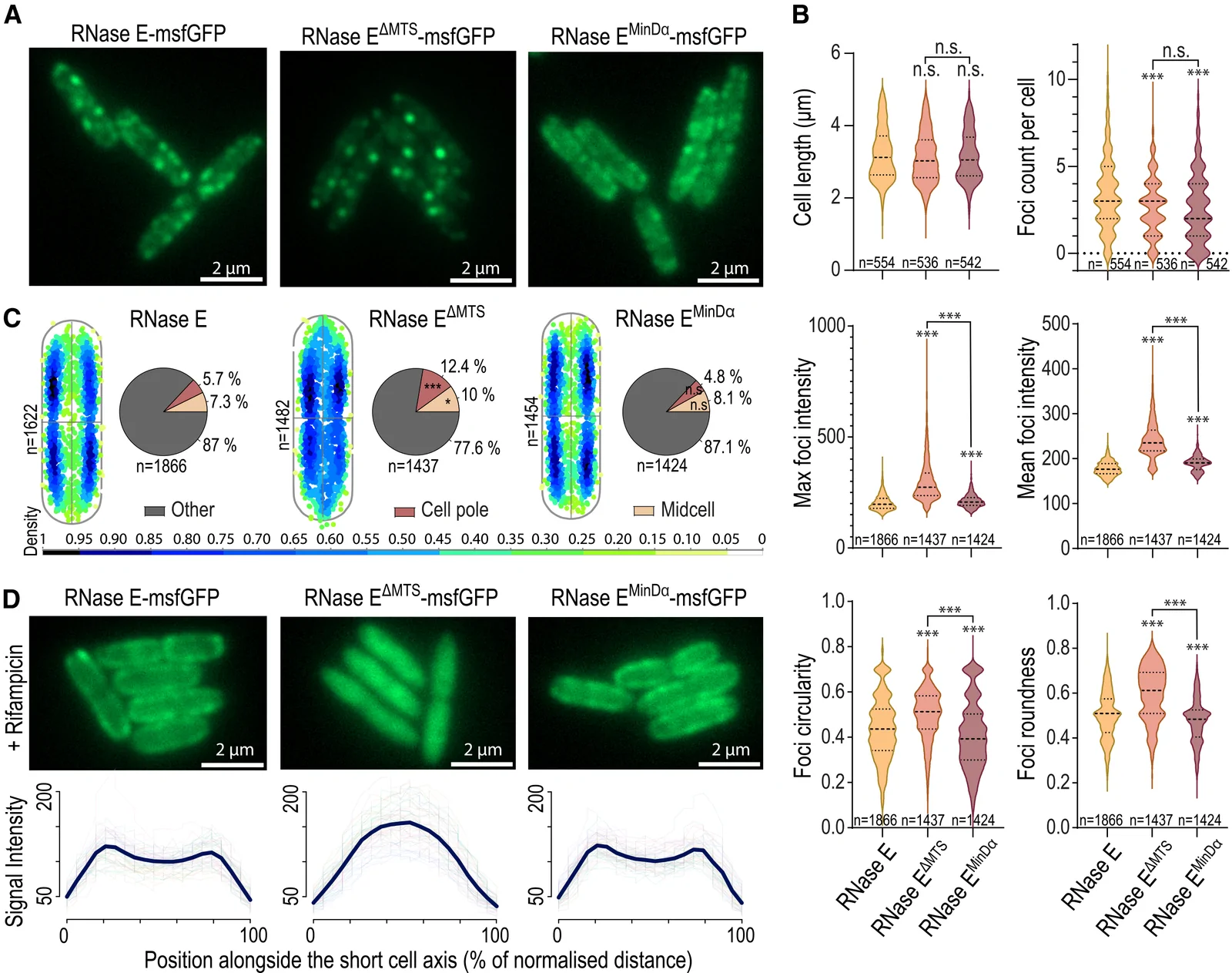
Deletion of the RNase E MTS causes foci morphology and localization defects which can be complemented by reinsertion of the unrelated MinDα (A) Representative images of RNase E, RNase E^ΔMTS^, or RNase E^MinDα^ C-terminally tagged with msfGFP (chromosomal expression from the native locus). (B) Violin plots (with median and quartiles) showing the quantification of cell length, RNase E foci count per cell, foci mean or max intensity, and foci circularity or roundness from images of msfGFP-tagged RNase E variants (as described) acquired in three independent biological replicates. The analyses were performed using the MicrobeJ^21^ plugin of Fiji^22^ as described in STAR Methods. An unpaired *t* test with Welch’s correction was done using GraphPad to compare mutant RNase E variants to the native RNase E (default comparison) or to each other (highlighted by brackets). n.s.: not significant (99% confidence interval); ∗∗∗: *p* value < 0.000001. (C) XY cell density graph of the average distribution of RNase E foci in a bacterial cell (left) and pie charts (right) highlighting the proportions of RNase E foci found at the midcell, the cell poles, or elsewhere (“Other”). Two-sample *χ*^2^ tests for equality of proportions with continuity correction were carried out. ∗: *p* value < 0.01; ∗∗∗: *p* value < 0.000001. Localization of the foci was automatically detected using the MicrobeJ^21^ plugin. The XY cell density plots were generated using MicrobeJ,^21^ whereby each detected focus, represented by a dot, is colored based on its vicinity with neighboring foci. For details about the settings used, see STAR Methods. (D) Representative images (top images) of RNase E, RNase E^ΔMTS^, or RNase E^MinDα^ C-terminally tagged with msfGFP (chromosomal expression from the native locus) after 30 min exposure to Rifampicin (100 μg/mL) and average fluorescence intensity profiles (bottom images) from the short cell axis of the rifampicin-exposed strains.

Although the primary sequence of the MTS varies across species, conserved hydrophobic or polar residues at defined positions maintain its amphipathic nature^19^ (Figure S1). To test whether this physicochemical property is sufficient for function, we constructed a chimera in which the amphipathic helix of the unrelated *E. coli* MinD protein^23^^,^^24^ (MinDα, Figure S1) was chromosomally inserted back at the native position in our strain expressing RNase E^ΔMTS^-msfGFP.^23^^,^^24^ Visualization of this strain expressing RNase E^MinDα^-msfGFP and automated quantification of foci morphology and localization revealed a nearly complete complementation to a WT-like appearance (Figures 1A–1C). Indeed, the average foci subcellular localization profile and counts at midcell or cell poles are virtually identical to those of full-length RNase E-msfGFP, while the foci are significantly less bright and less round than those of RNase E^ΔMTS^-msfGFP. Exposure of the strain expressing RNase E^MinDα^-msfGFP to rifampicin also results in a peripheral localization, with signal intensity profiles virtually identical to those of the full-length RNase E (Figure 1D). We also confirmed that protein levels of full-length RNase E, RNase E^ΔMTS^, and RNase E^MinDα^ are similar in exponentially growing cultures, ruling out altered protein abundance as the cause of the observed foci alterations (Figure S2).

Of note, we also generated two additional RNase E chimeric strains bearing two unrelated amphipathic helices (from *P. aeruginosa* PelC^25^ and *Mycoplasma genitalium* FtsZ^26^) that harbor several negative charges on their polar face: fluorescent signal visualization and quantification revealed a highly similar foci morphology and subcellular localization compared to the strain expressing RNase E^ΔMTS^-msfGFP (Figures S1, S3A, S3B, S3C, and S3D). These results suggest that not all amphipathic helices have features that can mediate anchoring to the inner membrane. The predicted net charges of PelCα and FtsZα are −1 and +2, respectively (Figure S1), indicating that overall net charge alone does not determine membrane association. We propose that the presence of negatively charged residues on the polar face of an amphipathic helix can cause electrostatic repulsion with phospholipids polar heads and impair membrane localization, consistent with the clear complementation obtained with the MinD amphipathic helix. Interestingly, the RNase E^MinDα^ foci are visually less well-defined, or more diffused, than those of the full-length RNase E (Figure 1A). Quantification of foci morphology confirms these observations and reveals significant differences in foci intensity and circularity/roundness compared to the full-length RNase E (Figure 1B), suggesting that intrinsic features of the amphipathic helix subtly regulate RNA degradosome clustering.

### RNase E MTS deletion causes stalling of the RNA degradosome foci and is partially rescued by MinDα

In *E. coli* and *C. crescentus*, RNase E foci are known to dynamically form and dissolve within the range of seconds.^8^^,^^11^ The altered morphology and increased intensity of RNase E^ΔMTS^ foci prompted us to investigate whether their assembly or dynamics were affected. To rule out an impaired recruitment of the RNA degradosome components PNPase and RhlB, we constructed a *P. aeruginosa* strain carrying both RhlB-msfGFP and PNPase-mTagBFP2 fusions at their native gene loci, together with a mCherry-tagged variant of RNase E (full-length, RNase E^ΔMTS^, or RNase E^MinDα^). Using structured illumination microscopy (SIM), we observed a strong colocalization of the three protein partners irrespective of the co-expressed RNase E variant (Figure 2), confirming that deletion of the MTS or its replacement with MinDα do not prevent recruitment of PNPase and RhlB into the RNase E-driven foci. Of note, the triple-tagged strain grows similarly to the WT strain, indicating that the three fluorescent fusions retain sufficient functionality to support normal growth (Figure S4, see also Geslain et al.^20^ for the single-tagged strains).

**Figure 2:**
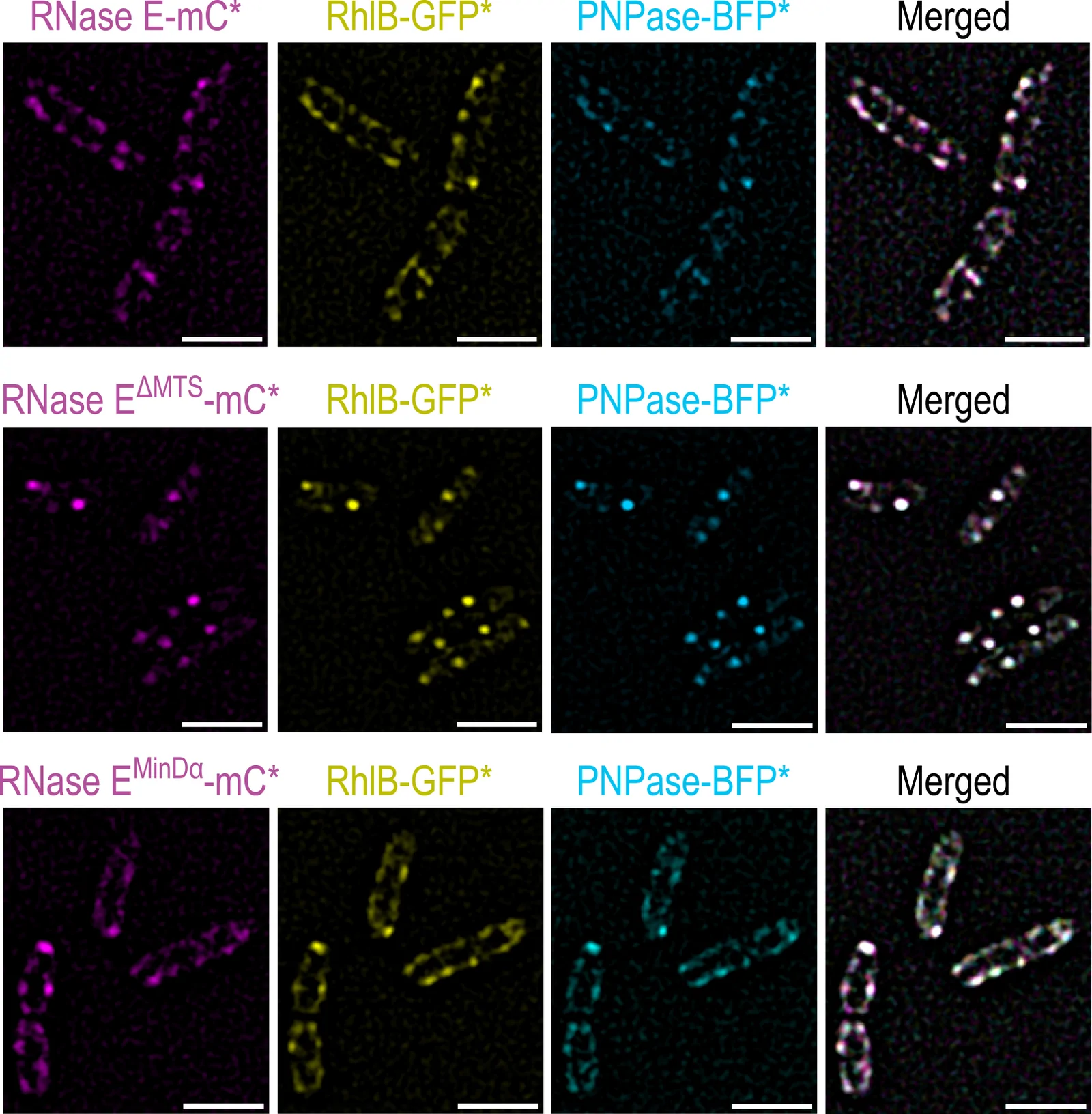
Deletion of the MTS or its replacement with MinDα does not impact recruitment of RhlB and PNPase into the RNase E-driven foci Visualization with structured illumination microscopy (Zeiss ELYRA7) of native or mutated RNase E-mCherry fusions as described (left column), RhlB-msfGFP fusion (middle column), or PNPase-mTagBFP2 fusion (second to last column) co-expressed in the same strain. mC∗: mCherry; GFP∗: msfGFP; BFP∗: mTagBFP2. The three channels were fused using Fiji^22^ to highlight signal overlap (Merged images). Scale is 2 μm.

We next used time-lapse SIM to investigate foci dynamics in the RNase E mutants. Strikingly, foci formed by RNase E^ΔMTS^ were severely stalled, with most remaining assembled and stationary throughout the 120-s acquisition period (Figure 3B; Video S2). This is in sharp contrast with foci assembled by RNase E full-length, which seem to rapidly dissolve and reassemble within the 5-s imaging frames, giving the impression that RNase E is circling through the bacterial membrane (Figure 3A; Video S1). Interestingly, we observed that the RNase E^MinDα^ foci seem to have an intermediate behavior: while most foci are dynamic and rapidly disappear and reform within 5 s, as observed with the full-length RNase E, some cells harbor several larger foci that remain stalled throughout the course of the time-lapse acquisition period (Figure 3C; Video S3).

**Figure 3:**
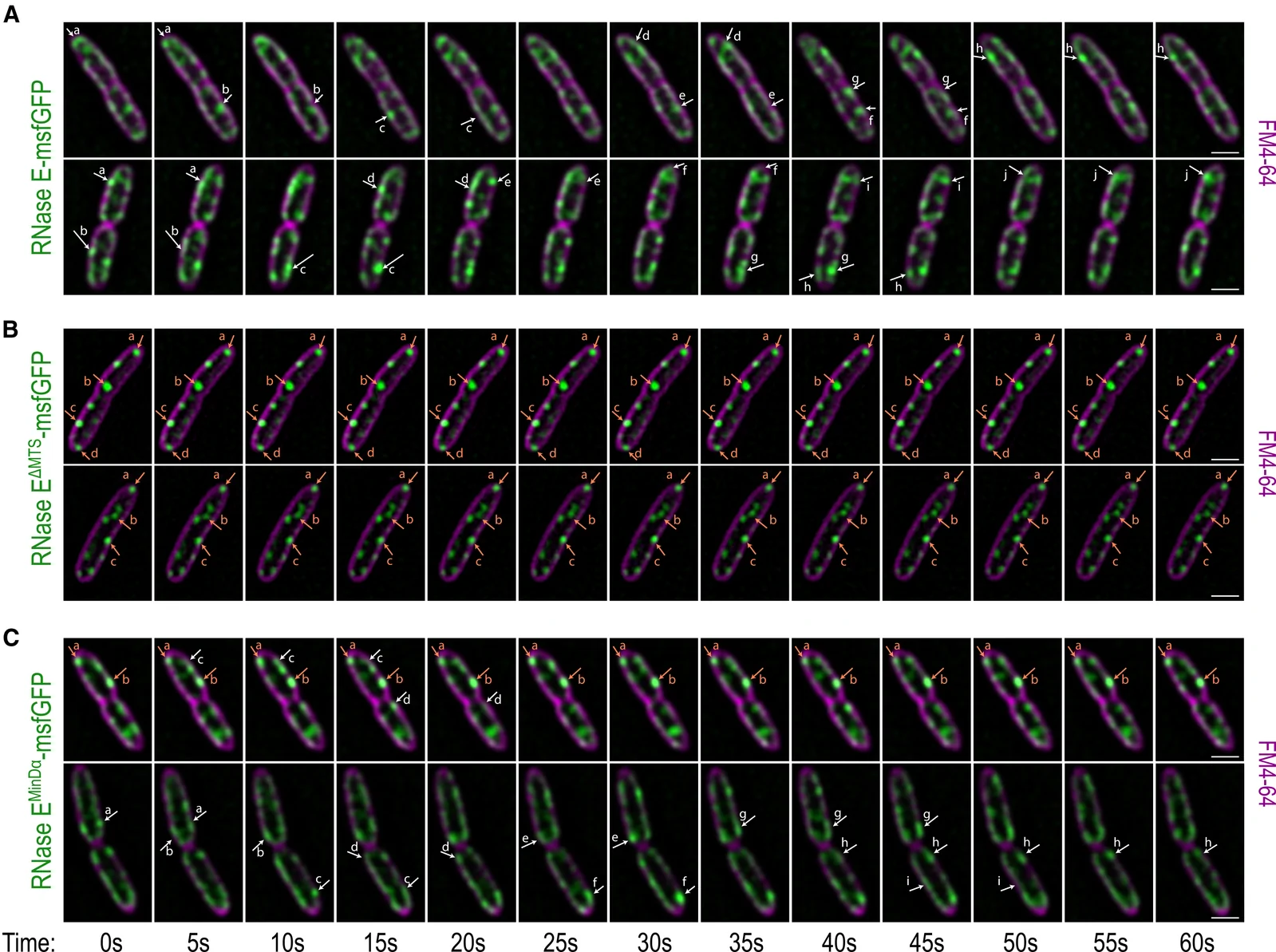
Loss of the MTS results in an immobilization of the RNase E foci, which can be rescued by reinsertion of MinDα SIM Time-lapse visualization of live bacterial cells chromosomally expressing RNase E-msfGFP (A), see also Video S1; RNase E^ΔMTS^-msfGFP (B), see also Video S2; or RNase E^MinDα^-msfGFP (C), see also Video S3. Membranes were stained using the FM4-64 dye. Foci that visually form, dissolve, or diffuse laterally are highlighted by white arrows, whereas foci that remain static throughout the acquisition are indicated by orange arrows. Letters indicate discrete foci. Scale is 1 μm. Channels showing the msfGFP and FM4-64 signal were merged for display using Fiji.^22^

Overall, these results suggest that the amphipathic nature of the MTS drives RNA degradosome localization and dynamics, whereas its specific sequence modulates foci behavior.

### The RNase E MTS is required for specific stress adaptation and can be substituted by MinDα

Despite the widespread conservation of the MTS in RNase E homologs, its functional importance for bacterial physiology and adaptive capacity remains unclear.^14^^,^^15^ To address this, we performed an extensive phenotypic characterization of the *rne*ΔMTS mutant, testing for growth on minimal medium or under acidic, basic, or ethanol stress conditions, as well as growth under exposure to detergents, salts, high temperatures, or iron-chelating agents (Figures 4 and S5). While the mutant grew nearly identically to the WT strain in rich medium at 37°C, with only minor growth defects observed at 16°C, it displayed a pronounced and specific growth defect in rich medium supplemented with 0.75 M NaCl or KCl, or 0.5 M (NH_4_)_2_SO_4_ (Figures 4A and S5). These defects were not attributable to osmotic stress alone: the *rne*ΔMTS mutant grew comparably to WT in medium supplemented with 0.75 M sucrose (Figure 4A). Together, these results indicate that deletion of the MTS confers a specific sensitivity to elevated ionic strength rather than to increased osmolarity per se.

**Figure 4:**
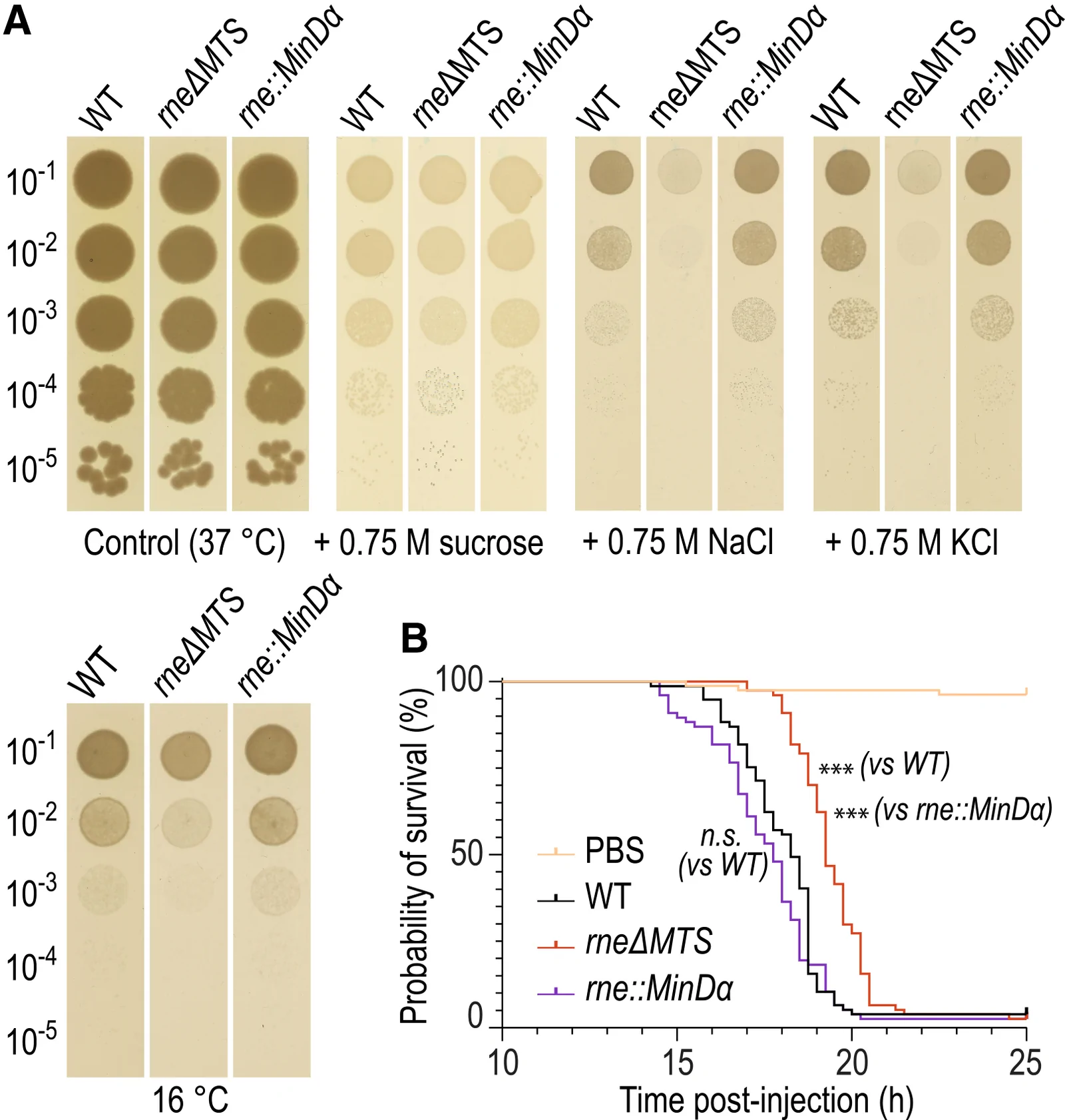
Reinsertion of MinDα can fully complement the phenotypic defects of the *rneΔMTS* strain in high salinity conditions or during infection of *Galleria mellonella* (A) Growth of PAO1 WT, *rne*ΔMTS, or *rne*:MinDα on solid medium at 37 °C under control conditions or with addition of 0.75 M NaCl, KCl, or sucrose to the growth medium. Growth at 16 °C was also tested (lower image). Three independent biological replicates were performed; a representative plate is shown. (B) Killing curves of *G. mellonella* larvae after injection with 10 μL of PAO1 WT, *rne*ΔMTS, or *rne*:MinDα dilutions. The control group was injected with 10 μL of sterile dPBS instead (see STAR Methods). Death was monitored over 14–25 h post-injection. Independent biological triplicates were done using 20–30 larvae per replicate (total of 77–80 larvae per strain tested). Statistical significance was assessed by performing a Kaplan-Meier survival analysis in GraphPad Prism using a log rank (Mantel-Cox) test and comparing the mutant RNase E variants to the native RNase E or to each other as described. ∗∗∗: *p* value < 0.0001; n.s.: not significant (99% confidence interval). Hazard ratios (log rank) and their reciprocals for the different pairwise comparisons are as follows: 2.271 and 0.4404 for WT versus *rne*ΔMTS; 0.7888 and 1.268 for WT versus *rne*:MinDα; 0.3910 and 2.558 for *rne*ΔMTS versus *rne*:MinDα.

To assess virulence, we performed a killing assay using *G. mellonella* larvae, an infection model highly susceptible to *P. aeruginosa* infections.^27^ Although near-complete mortality was observed by 24 h post-infection (*n* = 77 larvae), we detected a statistically significant delay of one to 2 h in killing time, indicating that the *rne*ΔMTS mutant is partially compromised in its ability to establish or sustain infection (Figure 4B). To assess whether the observed defects of the *rne*ΔMTS strain could be rescued by the *E. coli* MinDα, we reinserted MinDα back into the *rne*ΔMTS strain (at the native chromosomal position). Strikingly, the *rne*:MinDα strain can grow under stress or infect *G. mellonella* larvae virtually identically to the WT strain and is thus a full complementation of the *rne*ΔMTS defects (Figures 4A and 4B).

Overall, these results highlight the importance of the amphipathic nature of the helix for proper RNase E and RNA degradosome function under challenging conditions such as high salinity or host colonization.

### Deletion of RNase E MTS specifically stabilizes transcripts encoding proteins with transmembrane helices and impacts differential processing of some polycistronic transcripts

We previously performed transcriptomic analyses of two RNase E mutants lacking the C-terminal scaffolding domain, one mutant additionally lacking the MTS (*rne529*) and the other retaining it (*rne588*). Despite a comparable number of misregulated transcripts in the two mutants (400–450 transcripts in total), there was little overlap between the affected gene sets.^20^ This divergence prompted us to analyze the transcriptome of the *rne*ΔMTS mutant to clarify the specific contribution of the MTS to gene regulation. Capillary electrophoresis of total RNA samples revealed no difference in size profiles compared to WT, contrasting with previous reports in *E. coli* (Figure S6).^14^ Differential expression analysis identified only 93 significantly misregulated genes (Figure 5A), indicating that deletion of the MTS has a limited impact compared to the broader changes previously observed in the two aforementioned RNase E mutants. Nearly all upregulated transcripts and most downregulated transcripts in the *rne*ΔMTS mutant were similarly affected in the *rne529* mutant, supporting the conclusion that these changes are specifically attributable to loss of the MTS (Figure S7A). RT-qPCR analyses of five representative transcripts showed changes consistent with the RNA-seq results in direction and overall magnitude (Figure 5B). Gene Ontology (GO) enrichment analysis of the differentially regulated transcripts in the *rne*ΔMTS mutant further revealed overrepresentation of genes encoding transmembrane transporters as well as proteins involved in secondary metabolite biosynthesis or protein folding and proteolysis (Figure 5C).

**Figure 5:**
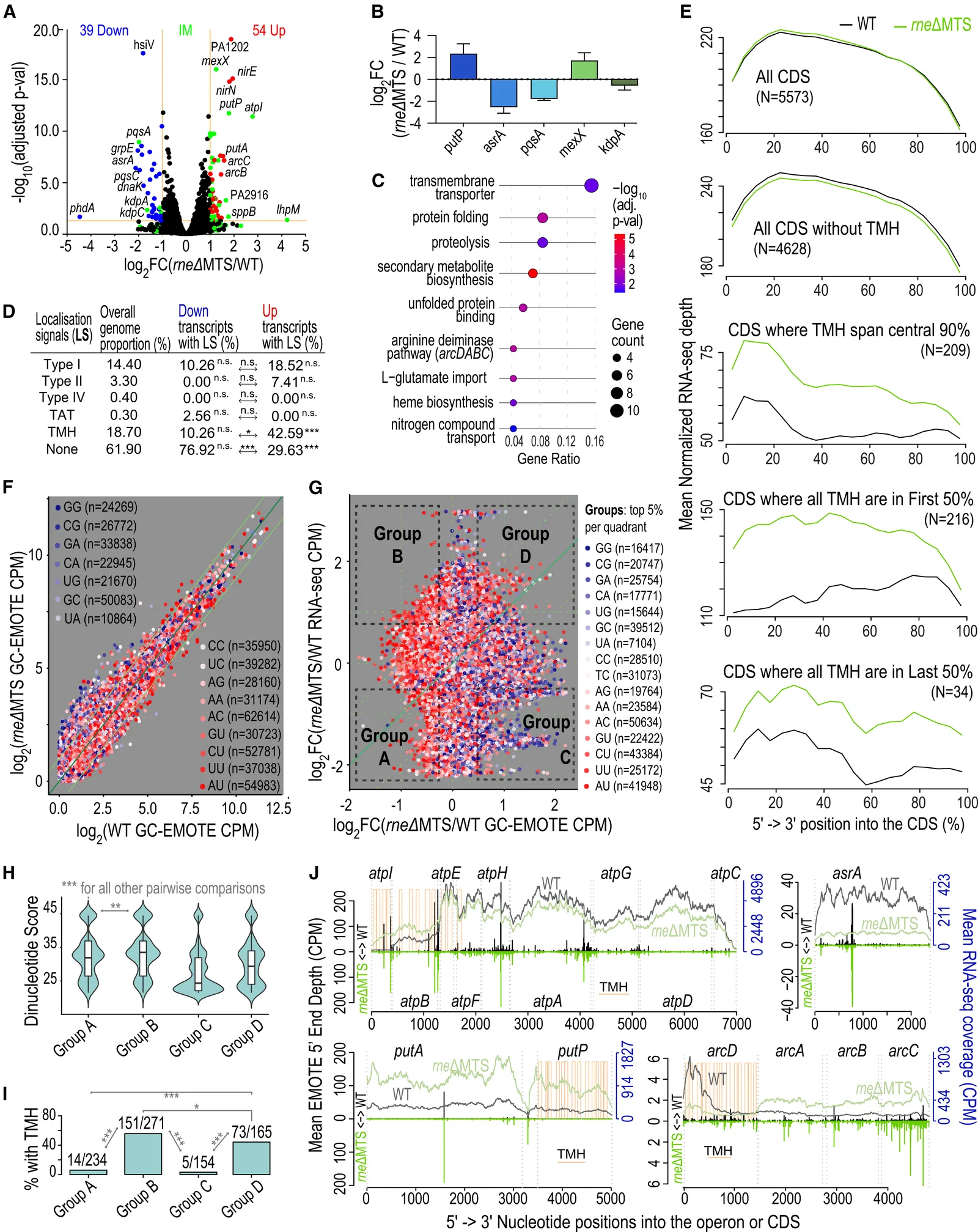
Systematic localized stabilization of TMH-encoding mRNAs in the *rne*ΔMTS mutant (RNA-seq) is linked to alterations in coverage depth at specific RNase E-compatible cleavage positions (GC-EMOTE) (A) Volcano plots highlighting genes upregulated (red) or downregulated (blue) in the *rne*ΔMTS mutant compared to the WT strain. Genes encoding inner membrane (IM) proteins are colored in green. Annotations of inner membrane proteins were retrieved from the Pseudomonas.com database. (B) RT-qPCR analysis of mean relative expression levels (fold change versus WT) with standard deviation of putP, asrA, pqsA, mexX, or kdpA transcripts in the *rneΔ*MTS. Relative expression levels were calculated as log_2_(2^−ΔΔCt^) using rpoD as an internal control. (C) Gene Ontology (GO) analysis of the RNA-seq dataset highlighting overrepresented gene functions misregulated in the *rne*ΔMTS mutant. (D) Table showing the proportions of downregulated (Down, blue) and upregulated (Up, red) transcripts from A whose encoded proteins contain a predicted localization signal (LS). LS annotations were retrieved from Lewenza et al.^28^ The overall genome proportion for each LS category was included as a comparison group, representing the expected reference. A two-sample *χ*^2^ test for equality of proportions with continuity correction was used to compare each set of transcripts with the corresponding genome proportion (default comparison, indicated next to each value) or to compare the downregulated and upregulated transcript sets with each other (indicated by arrows). n.s.: not significant; ∗: *p* value < 0.01 and ∗∗∗: *p* value < 0.0001. (E) RNA-Seq coverage metagenes from the WT (black) or *rneΔ*MTS (green) datasets for 5 groups of transcripts, hereafter described from top to bottom: 1. All CDS; 2. All CDS without TMH; 3. CDS where the first TMH is in the first 5% of the CDS and the last is in the final 95%; 4. CDS where the last TMH is in the first 50% of the CDS; 5. CDS where the first TMH is in the final 50% of the CDS. For each category of transcripts defined above, reads mapping within each CDS matching the aforementioned criteria were compiled. To account for variable gene lengths, each individual CDS was spatially normalized by partitioning it into exactly 20 equal-sized bins (representing 5% increments of the transcript length). For every transcript, the raw nucleotide coverage within each bin was averaged and subsequently normalized against the global library sequencing depth. This was done with coverages derived from pooled WT and *rne*ΔMTS replicates, respectively. (F) Scatterplot of GC-EMOTE 5′ end CPM depths in WT vs. *rne*ΔMTS. Each dot is the mean depth at a locus with at least one 5′ end in 2 or more WT or *rne*ΔMTS replicates. To evaluate the likelihood of a given 5′ end having been specifically generated by RNase E, each dot was colored based on compatibility with RNase E cleavage preferences of the dinucleotide at the 5′ end of the corresponding GC-EMOTE position-specific reads. GC-EMOTE 5′ ends with higher dinucleotide scores, represented in red shades, are very likely to have been generated by RNase E, whose preferred dinucleotide is WU (W = A, U). GC-EMOTE 5′ ends with lower dinucleotide scores, shown in shades of blue, widely differ from the consensus RNase E and either represent a 5′ end that arose from the activity of another endoribonuclease or an unusual RNase E cleavage site. Dinucleotide compatibility with RNase E cleavage was assessed as described in STAR Methods. (G) Plot of GC-EMOTE log2FC depth ratio *rne*ΔMTS/WT (all loci with at least one 5′ end in 2 or more WT or *rne*ΔMTS replicates) against the RNA-Seq log2FC *rne*ΔMTS/WT of the containing transcript. Four major quadrants, named group A, B, C, and D, are highlighted for reference and represent the ≈5% of transcripts that are most strongly up- or downregulated in the RNA-seq and/or the EMOTE dataset, respectively. The same dinucleotide compatibility score described in (F) was applied to sort GC-EMOTE 5′ ends found at a particular locus based on their likelihood to have been generated by RNase E (see STAR Methods). (H) Comparison of the RNase E compatibility dinucleotide score distributions for the GC-EMOTE 5′ end reads whose corresponding dot is found within one of the four quadrants illustrated in (G). A higher dinucleotide score indicates a higher likelihood that a given GC-EMOTE 5′ end has been generated by RNase E. Group A: *rne*ΔMTS/WT, GC-EMOTE bottom 5%, RNA-Seq bottom 5%. Group B: GC-EMOTE bottom 5%, RNA-Seq top 5%. Group C: GC-EMOTE top 5%, RNA-Seq top 5%. Group D: GC-EMOTE top 5%, RNA-Seq bottom 5%. Pairwise two-sided Welch’s *t* tests were conducted to compare the means of all possible group combinations, with significance indicated on the plots: ∗∗: *p* < 0.01; ∗∗∗: *p* < 0.0001. Cohen’s d effect sizes are −0.12 (A/B); 0.75 (A/C); 0.28 (A/D); 0.87 (B/C); 0.40 (B/D); and −0.45 (C/D). (I) Comparison of the percentage of CDSs encoding at least one TMH, within the A, B, C, and D groups illustrated in (G) and described in (H). Pairwise comparisons between groups were performed using two-sided Fisher’s exact tests; significance is shown on the plot: ∗: *p* < 0.05; ∗∗∗: *p* < 0.0001. Pairwise Cramer’s V effect sizes are 0.54 (A/B); 0.06 (A/C); 0.46 (A/D); 0.56 (B/C); 0.11 (B/D); and 0.49 (C/D). (J) GC-EMOTE peaks and RNA-seq coverage of one gene and three operons containing at least one transcript significantly misregulated in the *rne*ΔMTS transcriptomics analysis (A). The GC-EMOTE peaks found in the WT strain are shown in black (left y axis, upper half), while those found in the *rne*ΔMTS strain are displayed in green (left y axis, lower half). Relative height difference between black and green peaks is visually representative of differential peak depth between the two strains. The mean RNA-seq coverage (right y axis) is overlaid on the same graph as a dark gray line (WT strain) or a light green line (*rne*ΔMTS strain). The beginning and end of each CDS are highlighted by dotted light gray lines. The positions of transmembrane helices in the encoded protein are highlighted in light orange. TMH locations were predicted using DeepTMHMM. RNA-seq coverage shows differential transcript stabilization within the arc and the atp operons in the *rne*ΔMTS, correlating with varying GC-EMOTE peak depth between the two strains.

A previous study reported preferential stabilization of transcripts encoding inner membrane proteins in the *E. coli rne*ΔMTS strain.^3^ We therefore examined whether transcripts misregulated in the *P. aeruginosa rne*ΔMTS mutant encoded proteins with specific predicted subcellular localizations, using published annotations.^28^ Among the 54 upregulated transcripts, those encoding proteins with a predicted transmembrane helix (TMH) were significantly enriched (42.59%, *n* = 23), whereas transcripts lacking predicted localization signals and presumably encoding cytosolic proteins were underrepresented (29.63%, *n* = 16). Both proportions differed significantly from the corresponding overall proportions in the genome (18.70% and 61.90%, respectively) and from the proportions observed among downregulated transcripts (10.26% and 76.92%, respectively; Figure 5D). When compared with a control set of unchanged TMH-encoding transcripts, the upregulated set showed no difference in CDS length (Figure S7B) but was enriched for transcripts encoding proteins with more than six TMHs (Figure S7C). We therefore asked whether the increased abundance of TMH-encoding transcripts reflected preferential stabilization of their TMH-coding regions. Looking specifically at RNA-seq depth for reads mapping within all predicted TMH-encoding regions, we indeed observed a global, although modest, increase in read coverage in the *rne*ΔMTS, resulting in a clear shift in the coverage distribution (Figure S7D). Metagene analysis of RNA-seq coverage across CDS grouped according to their TMH profiles further showed that TMH-encoding transcripts are moderately but globally stabilized in the *rne*ΔMTS compared with CDS lacking TMHs. This effect was observed regardless of whether the TMHs were located in the first half, center or second half of the CDS (Figure 5E). Altogether, these findings support a model whereby RNase E membrane localization contributes to the preferential degradation of transcripts encoding proteins with multiple transmembrane helices.

To investigate how deletion of the MTS affected RNase E activity *in vivo*, we developed a modified version of 5’ monophosphorylated (5′ P) RNA sequencing, which we named GC-EMOTE.^29^ GC-EMOTE is an adaptation of the EMOTE (Exact Mapping Of Transcriptome Ends) protocol published by Redder,^29^ which we specifically tailored to GC-rich organisms through redesigned oligos and primers for 5’ end tagging, reverse transcription, and library amplification (Table S3; Figure S8). Additionally, we have improved several steps of library construction to enhance ligation efficiency and material recovery (see STAR Methods and Figure S8). A total of 563146 5’ P ends mapping to unique genomic positions were identified in the GC-EMOTE analysis. Their mean abundances in the *rne*ΔMTS and WT strains were compared in a scatterplot where each 5′ P end was colored according to the identity of its first two nucleotides (Figure 5F). Dinucleotides were scored and visually displayed from blue to dark red according to their compatibility with the established RNase E cleavage preference for WU immediately downstream of the cleavage site, where W is A or U.^30^^,^^31^^,^^32^^,^^33^^,^^34^^,^^35^ The most RNase E-compatible 5’ P ends (AU or UU dinucleotides, shown in dark red) were generally detected at high abundance in both strains, although a subset became specifically enriched in the *rne*ΔMTS mutant (Figures 5F and S7E). Poorly RNase E-compatible 5’ P ends were generally detected at lower abundance, although a small subset became enriched in the *rne*ΔMTS mutant (Figure S7E), potentially reflecting cleavage by another endoribonuclease.

To determine whether changes in GC-EMOTE 5′ end depth reflected changes in the overall abundance of the corresponding transcript or resulted from altered RNA processing at specific sites, we compared the relative GC-EMOTE depth of each 5′ end with the RNA-seq fold-change of its corresponding transcript in the *rne*ΔMTS mutant compared to WT. Data points falling within the most extreme 5% for both RNA-seq abundance and GC-EMOTE 5′ end depth in each direction were classified into four groups (A–D; Figure 5G). Group B, comprising transcripts most enriched in the *rne*ΔMTS RNA-seq while being most depleted at specific positions in the *rne*ΔMTS GC-EMOTE dataset, contains over 50% of TMH-encoding transcripts and is very likely to comprise RNase E targets based on our dinucleotide scoring (Group B, Figure 5G, 5H, and 5I). This suggess that loss of cleavage at specific sites, due to deletion of the MTS, results in transcript stabilization through a mechanism that preferentially impacts TMH-encoding transcripts. Conversely, the group of transcripts most depleted in the *rne*ΔMTS RNA-seq while being most enriched at specific positions in the *rne*ΔMTS GC-EMOTE dataset has almost no TMH-encoding transcripts and is depleted in RNase E target sites based on the dinucleotide scoring (Group C, Fig. 5GHI, see also Figure S7E). These results suggest a compensatory mechanism whereby, in the *rne*ΔMTS mutant, another ribonuclease—possibly RNase III—preferentially cleaves transcripts at poorly RNase E-compatible sites, thereby reducing the abundance of the corresponding transcripts.

To view how differential RNase E processing activity may contribute to gene misregulation, we analyzed the RNA-seq and GC-EMOTE coverage of operons and single genes affected in our transcriptome analysis (Figures 5J and S7F). Strikingly, genes found within the same polycistronic transcripts can have steady-state levels that are differentially altered in the *rne*ΔMTS mutant. For example, although the TMH-encoding transcripts arcD or atpI/atpB are respectively downregulated or upregulated in the *rne*ΔMTS mutant, most other genes in these operons follow the opposite pattern (Figure 5J). These alterations correlate with a GC-EMOTE profile showing different peak depth between the two strains. For example, the first peak found within atpI, whose overall transcript levels are very low in the WT strain, has a GC-EMOTE depth that is higher compared to the *rne*ΔMTS. We also observe one clear GC-EMOTE peak in the asrA transcript whose depth is greater in the *rne*ΔMTS despite a strong overall downregulation of transcript levels in the RNA-seq. Similarly, the TMH-encoding msbA and flhA are slightly upregulated in the *rne*ΔMTS mutant, while the downstream rfaE or flhF transcripts, respectively, are entirely unaffected. We interestingly observe the presence of a GC-EMOTE peak with high coverage that appears to overlap with the position on the transcript at which the stability shift occurs. These results suggest that our GC-EMOTE can confidently identify polycistronic RNA processing events which are ultimately responsible for differential transcript levels in both WT and *rne*ΔMTS strains, although we could not visually identify peaks that could instead explain the specific stabilization of the TMH-encoding transcript in the *rne*ΔMTS strain. Overall, our correlation of GC-EMOTE peak depth and RNA-seq coverage suggests that differential RNase E-mediated cleavage efficiency and/or cleavage site preferences account for the changes in polycistronic mRNA levels observed in the *rne*ΔMTS.

### Investigation of mRNA subcellular localization by smFISH reveals a specific membrane localization of a model TMH-encoding transcript stabilized in the rneΔMTS mutant

Because *E. coli* monocistronic or polycistronic mRNAs that encode at least one inner membrane protein are known to themselves locate near the inner membrane,^3^^,^^4^^,^^36^ we hypothesized that most of the TMH-encoding transcripts upregulated in the *rne*ΔMTS genetic background become shielded from the RNA degradosome due to their segregation at the inner membrane. Choosing three well-expressed transcripts showing different patterns in the RNA-seq, namely asrA (cytosolic protease; downregulated), pqsAB (major quorum sensing system; PqsA: putative inner membrane protein but lacking predicted TMH, PqsB: cytosolic; both downregulated) and putP (inner membrane proline/sodium symporter; upregulated), we specifically investigated mRNA subcellular localization in *P. aeruginosa* using non-multiplexed single-molecule RNA Fluorescence *In Situ* Hybridisation (smFISH).

Epifluorescence imaging of smFISH-treated bacterial samples revealed discrete fluorescent foci in both the WT and *rne*ΔMTS strains for all three genes tested (Figures 6A and S9). In smFISH, a single mRNA molecule typically appears as one bright, diffraction-limited focus due to signal amplification from the annealing of approximately 48 fluorescent probes per transcript.^37^ To verify probe specificity, we generated deletion mutants for each of the three genes and confirmed that the signal observed in the WT and *rne*ΔMTS strains was specific to the target mRNAs (Figures 6A and S9). Automated quantification of the smFISH images showed that differences in foci number and intensity between WT and *rne*ΔMTS mirrored the RNA-seq results. Indeed, the upregulated putP transcript displayed increased foci counts and higher signal intensity in the *rne*ΔMTS strain, whereas the downregulated asrA and pqsAB transcripts exhibited reduced foci numbers and intensities compared to the WT (Figures 6C and 6D). Although a few foci were automatically detected in the deletion mutants, their fluorescence intensity was minimal and consistent with background signal, and no foci could be visually observed.

**Figure 6:**
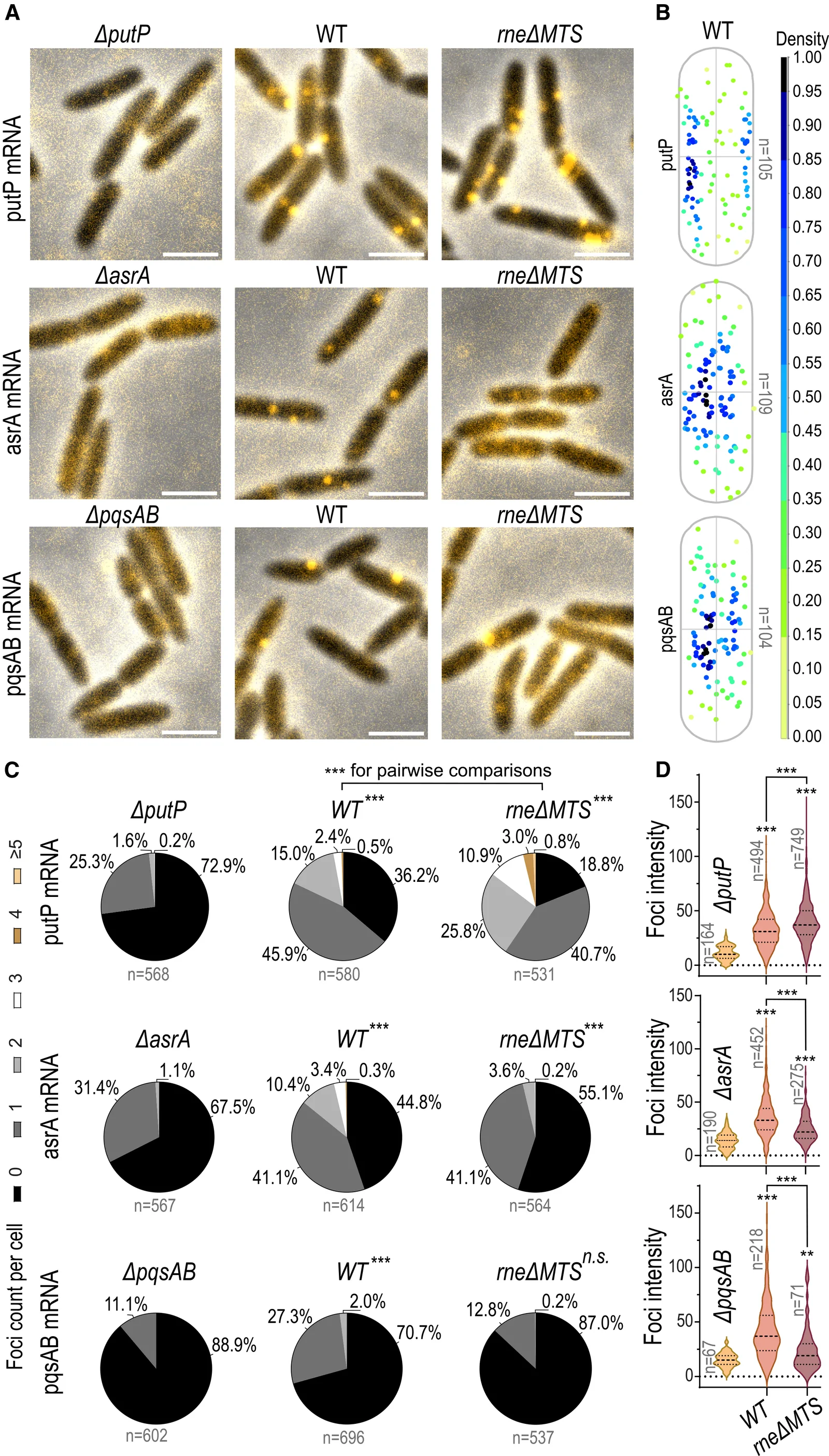
Single-molecule fluorescence *in situ* hybridization (smFISH) reveals different overall localization patterns of the putP, pqsAB, and asrA transcripts and confirms altered expression levels in the rneΔMTS strain (A and B) Representative images (A) and XY cell density graph of the average distribution of FISH foci (B) for the putP (upper images), asrA (middle images), or pqsAB (lower images) mRNAs. One foci theoretically corresponds to one mRNA molecule; *n* values correspond to the number of foci detected. Scale is 2 μm. The XY cell density graph of the average distribution of FISH foci was generated with MicrobeJ^21^ using three images for each transcript, each acquired in independent biological replicates. (C and D) Pie charts (C) and violin plots with median and quartiles (D) highlighting the proportions of cells having 0, 1, or more foci of putP (upper images), asrA (middle images), or pqsAB (lower images) mRNA and the measured intensity of those foci, respectively. The foci detection and intensity measurement were automatically done with the MicrobeJ^21^ plugin in Fiji^22^ using images acquired in three independent biological replicates. One to three images were analyzed for each biological replicate (approximately 125–250 cells per biological replicate). A two-sample *χ*^2^ test for equality of proportions with continuity correction was used to compare the proportions of foci per cell (C) in the WT or *rne*ΔMTS strain relative to the negative control (default comparison) or relative to one another (highlighted by brackets). ∗∗∗: *p* value < 0.0001; n.s.: not significant. Unpaired *t* tests with Welch correction were performed using GraphPad to compare the foci intensities (D) observed in the WT or *rne*ΔMTS strain relative to the negative control (default comparison) or relative to one another (highlighted by brackets). ∗∗∗: *p* value < 0.000001 and ∗∗: *p* value < 0.001.

We next investigated the subcellular distribution of putP, asrA, and pqsAB mRNA foci using stringent detection parameters (Figure 6B). The distribution profiles reveal that putP mRNAs are preferentially enriched near the membrane, consistent with the localization of the encoded protein and with its stabilization in the *rne*ΔMTS strain. Conversely, asrA mRNAs, which encode a cytosolic protein, are distributed throughout the cytoplasm but appear enriched toward the center of the cell. Although individual pqsAB mRNA foci were observed near the membrane, the overall distribution profile suggests a ubiquitous localization, with signal also concentrated near the center of the cell. The observed pqsAB mRNA distribution indicates limited membrane enrichment, consistent with the downregulation of the pqsABCDE operon in the *rne*ΔMTS strain. Alternatively, processing of the pqsABCDE polycistronic mRNA may generate truncated transcripts of varying length showing distinct localization patterns, potentially accounting for the mixed distribution observed.

Overall, our smFISH images reveal distinct subcellular localization patterns for putP, asrA, and pqsAB mRNAs and provide independent support for their consistent differential regulation in the *rne*ΔMTS strain, highlighting an intricate connection between mRNA localization and RNA degradosome segregation.

## Discussion

The interplay between RNA degradosome compartmentation and mRNA localization has recently emerged as a core element influencing gene expression and mRNA stability.^1^^,^^3^^,^^17^ However, our understanding of the means through which bacteria spatially organize the RNA degradosome, as well as the biological consequences of this compartmentation, remains limited to a few case studies.

One key feature which determines the subcellular localization of Gammaproteobacterial RNA degradosomes is the conserved RNase E membrane-targeting sequence (MTS). In the present study, we show that a *P. aeruginosa* RNase E mutant lacking the MTS is partially delocalized from the membrane. In the *rne*ΔMTS mutant, a broader RNase E subcellular distribution compared to the WT is observed, with a concomitant increase in the fraction of foci localizing at the cell poles or at the midcell. Importantly, in the *rne*ΔMTS mutant, the RNA degradosome foci are still assembled, but they have abnormal characteristics: foci are significantly larger, more rounded, and have an increased signal intensity compared to WT foci. Time-lapse SIM revealed that these foci are also severely stalled and cannot rapidly dissolve. Overall, these results suggest that deletion of the MTS in *P. aeruginosa* RNase E modifies the physical properties and dynamics of RNase E-driven biomolecular assemblies without abolishing their formation, thus maintaining a degree of RNA degradosome compartmentation.

Contrastingly, previous studies performed in *E. coli* showed that deletion of the MTS not only results in delocalization of RNase E from the membrane but also completely abolishes foci formation, ultimately resulting in an increased RNase E diffusion coefficient in the *E. coli rne*ΔMTS compared to the WT.^11^^,^^12^^,^^13^ These results highlight the necessity of membrane anchoring for foci assembly or stability in this organism. The difference between *E. coli* and *P. aeruginosa* MTS deletion mutants likely arises from species-specific properties of the RNase E C-terminal scaffolding domain.^19^ In a previous study, we described a REER-repeats region within the *P. aeruginosa* RNase E C-terminal domain that is highly rich in arginine (R) and glutamate (E) residues.^20^ This region, which is necessary for foci formation and binds RNA, is completely absent from the *E. coli* RNase E homolog.^19^ Interestingly, alphaproteobacterial RNase E homologs do not contain an MTS but drive the formation of biomolecular condensates owing to alternating blocks of negatively or positively charged residues,^8^ suggesting that the REER-repeats region may fulfill a similar function in *P. aeruginosa* and bypass the need of the MTS for maintenance of RNA degradosome compartmentation.

Consistent with the different effect of the RNase E MTS deletion on RNA degradosome organization between *E. coli* and *P. aeruginosa*, distinct transcriptome profiles are observed in the two species. In *E. coli*, loss of the MTS results in widespread mRNA stabilization affecting more than 240 transcripts,^3^^,^^15^ indicating a broad disruption of RNA turnover. In contrast, the *P. aeruginosa rne*ΔMTS mutant displays a relatively limited set of misregulated transcripts at steady state (*n* = 93), suggesting that RNA degradosome compartmentation and its associated enzymatic activity are only partially affected.

Correlating the RNA-seq dataset with GC-EMOTE 5′ P counts, we show that the subset of transcripts that are most upregulated in the *rne*ΔMTS mutant RNA-seq often encode transmembrane helices and also have a loss of 5′ P end coverage at sites that, based on scoring of the dinucleotide located at the cleavage position, are likely RNase E targets. Our investigation of GC-EMOTE 5′P counts and RNA-seq coverage mapping within polycistronic transcripts additionally revealed specific alterations in the relative abundance between different CDS within operons containing one TMH-encoding transcript. Although specific changes in translation rates and ribosome traffic may also result in differential stabilization within polycistronic transcripts due to shielding or exposing of mRNA segments, our results strongly suggest that deletion of the MTS impairs the regulatory RNase E-mediated processing of some polycistronic transcripts. Altogether, our coupled RNA-seq/GC-EMOTE analysis reveals that a large group of transmembrane helix-encoding transcripts are stabilized in the *rne*ΔMTS mutant, presumably due to decreased recognition by RNase E. Preferential stabilization of inner membrane protein-encoding transcripts upon deletion of the RNase E MTS has also been reported in *E. coli*,^3^ leading the authors to propose a model in which co-translational insertion of nascent inner membrane proteins localizes their mRNAs near the membrane, in the vicinity of RNase E.^3^ Deletion of the RNase E MTS disrupts this spatial coupling and results in selective stabilization of transcripts normally more rapidly decayed at the membrane. We hypothesize that a comparable mechanism may occur in *P. aeruginosa* and confirmed using three model transcripts that mRNAs encoding TMH-containing proteins indeed appear to localize near the inner membrane in this bacterium, while transcripts encoding cytosolic proteins do not.

Beyond an impact on transcript levels, our study demonstrates that altered RNA degradosome organization leads to specific phenotypic impairments in *P. aeruginosa*. Deletion of the RNase E MTS results in a pronounced growth defect under hypersaline conditions induced by NaCl or KCl, whereas sucrose does not impair growth relative to the WT strain, suggesting a sensitivity to ionic stress rather than osmotic pressure per se. The mutant also exhibits mild cold sensitivity, a phenotype commonly associated with mutations in core RNA-binding proteins.^38^^,^^39^^,^^40^ Moreover, the modest but reproducible attenuation of virulence further implicates MTS-dependent RNA degradosome spatial organization in host adaptation. These phenotypes may arise from disrupted processing of membrane-localized mRNAs in the MTS-deleted background. Alternatively, redistribution of RNase E within the cell may bring it into contact with RNA populations that are normally spatially segregated from the RNA degradosome, resulting in inappropriate RNA processing or decay and impaired stress adaptation. In *E. coli*, various small RNAs become strongly enriched at the cell poles during osmotic stress, suggesting that the partial polar relocalization of RNase E observed in the *rne*ΔMTS mutant could perturb their expression levels.^4^ Altogether, these findings establish a direct functional link between RNA degradosome spatial organization and bacterial stress adaptation.

To determine whether the MTS-mediated control of RNA degradosome foci assembly, dynamics, and subcellular localization depends primarily on amphipathicity or on specific sequence features within the MTS, we replaced the native MTS with a heterologous amphipathic helix. This approach allowed us to experimentally distinguish phenotypes caused by mere loss of amphipathicity from sequence-specific effects. The presence of an unrelated positively charged amphipathic helix was sufficient to restore RNase E membrane localization, re-establish RNA degradosome organization into transient foci, and rescue stress adaptation. However, incomplete restoration of foci morphology and dynamics in the chimeric strain indicates that the native MTS sequence possesses intrinsic features which can modulate RNA degradosome behavior beyond mere membrane targeting. Differences in primary sequence may influence the strength or stability of membrane association, thereby affecting spatial compartmentation and higher-order organization of the RNA degradosome. Alternatively, the MTS may also participate in regulatory protein-protein interactions. In *E. coli*, the oscillating protein MinD transiently interacts with the RNase E MTS; its deletion results in frequent polar accumulation of RNase E and a significant impairment of RNase E dynamics,^17^ echoing some of the alterations we observed in the *P. aeruginosa rne*ΔMTS mutant.

Overall, our study establishes the RNase E MTS as a multifunctional determinant of RNA degradosome organization in *P. aeruginosa*. Beyond acting as a membrane anchor, the MTS modulates bacterial stress adaptation and virulence through its control of RNA degradosome subcellular distribution, dynamics, and mRNA processing and steady-state levels. Our findings demonstrate that amphipathic helices are not mere anchors and can encode regulatory information. More broadly, our study highlights subcellular organization as a fundamental layer of bacterial gene regulation and illustrates how cellular architecture shapes adaptive gene expression.

### Limitations of the study

Our investigation of phenotypic defects in the *rneΔMTS* mutant revealed a moderate but significant virulence defect in a *G. mellonella* model, observable as a delay in the killing curve. Future studies in mammalian infection models will be needed to determine whether these virulence and environmental adaptation defects also occur in more complex host environments.^41^ In our coupled RNA-seq and GC-EMOTE analyses, we sorted 5′ P ends based on their compatibility with the RNase E cleavage site consensus sequence determined in *S. enterica*, *E. coli,* and other bacterial species. Although the *P. aeruginosa* RNase E catalytic domain (residues 1–529) is structurally conserved and has 70% identity with the *E. coli* homolog, we cannot exclude that minor differences in catalytic activity and sequence preference exist between the *P. aeruginosa* and the *E. coli* enzymes. Finally, our FISH analysis of three representative transcripts was highly consistent with our transcriptomic results but could benefit from being extended to a greater number of transcripts coding for differently localized proteins to confirm the observed trend of preferential stabilization of membrane-localized transcripts.

## Resource availability

### Lead contact

Requests for further information and resources should be directed to and will be fulfilled by the lead contact, Martina Valentini (martina.valentini@unige.ch).

### Materials availability

All strains and plasmids will be provided upon request to the lead contact.

### Data and code availability

• RNA-seq and GC-EMOTE data have been deposited at GEO and are publicly available as of the date of publication at GEO: GSE330145. All original, unprocessed data have been deposited at Mendeley as of the date of publication at https://doi.org/10.17632/x6bmfnpymb.2.
• All original code has been deposited at Zenodo and is publicly available at https://doi.org/10.5281/zenodo.21648181 as of the date of publication.
• Any additional information required to reanalyze the data reported in this paper is available from the lead contact upon request.

## Acknowledgments

We would like to thank Dr. Christoph Engl (Queen Mary University of London) for advice concerning the FISH protocol. This work was supported by a 10.13039/501100001711Swiss National Science Foundation Eccellenza grant (PCEFP3_203343, to M.V.) and additionally funded by the 10.13039/501100023177Fondation Pierre Mercier pour la Science (to M.V.) and the 10.13039/501100016199iGE3 PhD Salary awards 2024 (to S.A.M.G).

## Author contributions

Conceptualization, S.A.M.G. and M.V.; data curation S.A.M.G., G.E.A., and M.V.; formal analysis S.A.M.G., G.E.A., and M.V.; methodology, S.A.M.G., G.E.A., P.R., and M.V.; investigation, S.A.M.G., J.G., G.E.A., and M.V.; project administration M.V.; resources S.A.M.G, J.G., and M.V.; software G.E.A.; validation S.A.M.G., J.G., G.E.A., and M.V.; visualization S.A.M.G., G.E.A., and M.V.; writing – original draft S.A.M.G. and M.V.; writing – review and editing, S.A.M.G, G.E.A, J.G., P.R., and M.V.; funding acquisition, S.A.M.G. and M.V.; supervision, P.R. and M.V.

## Declaration of interests

The authors declare no competing interests.

## STAR★Methods

### Key resources table

**Table:** 

REAGENT or RESOURCE	SOURCE	IDENTIFIER
**Antibodies**		

Anti-mouse IgG (whole molecule)-peroxidase antibody produced in rabbit	Sigma-Aldrich	Cat# A9044-2mL
Anti-rabbit IgG (whole molecule)-peroxidase antibody produced in goat	Sigma-Aldrich	Cat# A6154-1mL
StrepMAB-Classic Monoclonal antibody	Iba Lifesciences	Cat# 2–1057-001
Pseudomonas aeruginosa oprF polyclonal antibody	ThermoFisher	Cat# PA5-117553

**Bacterial and virus strains**		

*Escherichia coli* DH5α	Lab collection	N/A
*Escherichia coli* HB101 (pRK2013)	Lab collection	N/A
*Pseudomonas aeruginosa* PAO1	Lab collection	WT
PAO1 *rne::2xStrep*	Geslain et al.^20^; chromosomal insertion of a Twin Strep-tag immediately upstream of the stop codon in *rne*	N/A
PAO1 *rne::msfGFP*	Geslain et al.^20^; chromosomal insertion of *msfGFP* with a flexible linker immediately upstream of the stop codon in *rne*	N/A
PAO1 *rneΔMTS*	This study; in-frame deletion of *rne* sequence coding for residues 552–573 (MTS and a few downstream residues) in the PAO1 WT strain	N/A
PAO1 *rneΔMTS::2xStrep*	This study; in-frame deletion of *rne* sequence coding for residues 552–573 (MTS and a few downstream residues) in the PAO1 *rne*::2xStrep strain	N/A
PAO1 *rneΔMTS::msfGFP*	This study; in-frame deletion of *rne* sequence coding for residues 552–573 (MTS and a few downstream residues) in the PAO1 *rne*::msfGFP strain	N/A
PAO1 *rneMinDα*	This study; in-frame insertion of the sequence coding for *E. coli* MinD amphipathic helix (MinDα) in the PAO1 *rneΔMTS* at the native MTS position	N/A
PAO1 *rneMinDα::2xStrep*	This study; in-frame insertion of the sequence coding for *E. coli* MinD amphipathic helix (MinDα) in the PAO1 *rneΔMTS::2xStrep* at the native MTS position	N/A
PAO1 *rneMinDα::msfGFP*	This study; in-frame insertion of the sequence coding for *E. coli* MinD amphipathic helix (MinDα) in the PAO1 *rneΔMTS::msfGFP* at the native MTS position	N/A
PAO1 *rnePelCα::msfGFP*	This study; in-frame insertion of the sequence coding for *P. aeruginosa* PelC amphipathic helix (PelCα) in the PAO1 *rneΔMTS::msfGFP* at the native MTS position	N/A
PAO1 *rneFtsZα::msfGFP*	This study; in-frame insertion of the sequence coding for *M. genitalium* FtsZ amphipathic helix (FtsZα) in the PAO1 *rneΔMTS::msfGFP* at the native MTS position	N/A
PAO1 *rhl::msfGFP*/*rne::mCherry*/*pnp::mTagBFP2*	This study; chromosomal insertion of *mTagBFP2* with a flexible linker immediately upstream of the *pnp* stop codon in the strain PAO1 *rhl*::*msfGFP*/*rne*::*mCherry* (from Geslain et al.^20^).	N/A
PAO1 *rhl::msfGFP*/*rneΔMTS::mCherry*/*pnp::mTagBFP2*	This study; deletion of the *rne* sequence coding for residues 552–573 (MTS and a few neighboring residues) in the PAO1 *rhl::msfGFP*/*rne::mCherry*/*pnp::mTagBFP2* strain	N/A
PAO1 *rhl::msfGFP*/*rneMinDα::mCherry*/*pnp::mTagBFP2*	This study; in-frame insertion of the sequence coding for *E. coli* MinD amphipathic helix (MinDα) in the PAO1 *rhl::msfGFP*/*rneΔMTS::mCherry*/*pnp::mTagBFP2* strain at the native MTS position	N/A
PAO1 *ΔputP*	This study; deletion of the entire *putP* CDS and partial deletion of intergenic regions or UTRs (genomic positions 855224–856801)	N/A
PAO1 *ΔasrA*	This study; in-frame deletion of most of the *asrA* CDS (genomic positions 845820–848183)	N/A
PAO1 *ΔpqsAB*	This study; in-frame deletion of most of the *pqsA* and *pqsB* CDS (genomic positions 1078468–1080785)	N/A

**Chemicals, peptides, and recombinant proteins**		

Nutrient broth	Condalab	Cat# 1216
Bacto Yeast extract	Gibco, ThermoFisher	Cat# 212750
Blood Agar Base	Merck Millipore	Cat# 70133-500G
BD Difco Luria Bertani (LB) LB broth, Miller	ThermoFisher	Cat# DF0446-17-3
LB Agar - Powder according to Miller	PanReac Applichem	Cat# A0927
LB Lennox Broth base	Invitrogen, ThermoFisher	Cat# 12780052
European Bacteriological Agar	Condalab	Cat# 1800
Bacto Tryptone	Gibco, ThermoFisher	Cat# 211705
Tetracycline	Sigma-Aldrich	Cat# 87128-25G
Gentamicin sulfate	Sigma-Aldrich	Cat# G1914-5G
Kanamycin sulfate	PanReac AppliChem	Cat# A1493,0010
Chloramphenicol	Sigma-Aldrich	Cat# C0378-25G
Carbenicillin disodium salt	Sigma-Aldrich	Cat# C1389-5G
Lysozyme	PanReac AppliChem	Cat# A3711,0001
Q5 High-Fidelity DNA polymerase	New England Biolabs	Cat# M0491S
UltraPure DNase/RNase-free Distilled Water	Invitrogen, ThermoFisher	Cat# 10977049
NEBuilder HiFi DNA assembly Master Mix	New England Biolabs	Cat# E2621L
GoTaq DNA polymerase	Promega	Cat# M3001
FM4-64	Invitrogen, ThermoFisher	Cat# T13320
UltraPure Low Melting Point Agarose	Invitrogen, ThermoFisher	Cat# 16520050
Dulbecco’s Phosphate-Buffered Saline (dPBS)	Gibco, ThermoFisher	Cat# 14040–091
RNAlater	Sigma-Aldrich	Cat# R0901-500ML
TURBO DNase	Invitrogen, ThermoFisher	Cat# AM2238
GlycoBlue Coprecipitant	Invitrogen, ThermoFisher	Cat# AM9515
T4 RNA ligase 1	New England Biolabs	Cat# M0204S
RNasin Plus Ribonuclease Inhibitor	Promega	Cat# N2611
Tris-EDTA (TE) buffer pH 8.0, RNase-free	Invitrogen, ThermoFisher	Cat# AM9849
ImProm-II Reverse Transcriptase	Promega	Cat# A3802
AMPure XP Beads for DNA Cleanup	Beckman Coulter	Cat# A63881
Formaldehyde 36.5–38%	Sigma-Aldrich	Cat# F8775-500ML
10*X* PBS buffer pH 7.4, RNase-free	Invitrogen, ThermoFisher	Cat# AM9624
3M Sodium acetate pH 5.5, RNase-free	Invitrogen, ThermoFisher	Cat# AM9740
Ribonucleoside Vanadyl Complex	New England Biolabs	Cat# S1402S
Dextran sulfate sodium salt from Leuconostoc spp.	Sigma-Aldrich	Cat# D8906-10G
tRNA from baker’s yeast (*S. cerevisiae*)	Sigma-Aldrich	Cat# R8759-500UN
Formamide	Invitrogen, ThermoFisher	Cat# AM9342
20*X* Saline Sodium Citrate (SSC) buffer	Invitrogen, ThermoFisher	Cat# AM9770
Ultrapure Bovine Serum Albumin (BSA)	Invitrogen, ThermoFisher	Cat# AM2616
TopVision Low Melting Point Agarose	ThermoFisher Scientific	Cat# R0801
Crystal Violet	Sigma-Aldrich	Cat# 61135-25G

**Critical commercial assays**		

Monarch Total RNA Miniprep kit	New England Biolab	Cat# T2010S
riboPOOL kit for *P. aeruginosa*	siTOOLS Biotech	N/A
SensiFAST SYBR Hi-ROX Kit	Bioline	Cat# BIO-92005
SuperSignal West Pico PLUS Chemiluminescent Substrate	ThermoFisher	Cat# 34577

**Deposited data**		

RNA-sequencing and GC-EMOTE data	This study, NCBI GEO	GEO: GSE330145
Raw data	This study, Mendeley data	Mendeley data: https://doi.org/10.17632/x6bmfnpymb.2
Original code	This study, Zenodo	Zenodo: https://doi.org/10.5281/zenodo.21648181

**Experimental models: Organisms/strains**		

*Galleria mellonella*	Bait Express GmbH	N/A

**Oligonucleotides**		

See Table S1 (Primers)	N/A	N/A
See Table S3 (GC-EMOTE oligos and primers)	N/A	N/A
See Table S4 (FISH probes)	Stellaris	N/A

**Recombinant DNA**		

See Table S2 (Plasmids table)	N/A	N/A

**Software and algorithms**		

Fiji 2.16	Open Source^22^	https://fiji.sc/; RRID: SCR_002285
Fiji plugin MicrobeJ 5.13p	Brun lab^21^	https://brunlab.com/research/microbej/; RRID: SCR_023914
GraphPad Prism 10.3.1	GraphPad Software	https://www.graphpad.com/; RRID: SCR_002798
Zen 3.5 (blue edition)	Zeiss	https://www.zeiss.com/microscopy/en/products/software/zeiss-zen.html; RRID: SCR_013672
Snapgene 8.0.3	Dotmatics	https://www.snapgene.com/; RRID: SCR_015052
Stellaris RNA FISH probe designer	LGC BioSearch Technologies	https://oligos.biosearchtech.com/support/resources/stellaris-rna-fish; RRID: N/A
PerlPrimer	Open Source^42^	https://perlprimer.sourceforge.net/; RRID: SCR_012038
R	Open Source^43^	https://www.R-project.org/; RRID: SCR_001905

**Other**		

Omnican 50 sterile insulin syringe	Braun	9151125S

### Experimental model and study participant details

#### Bacterial strains and growth conditions

*Pseudomonas aeruginosa* PAO1 has been used as an experimental model throughout this study.

*Escherichia coli* DH5α was used for cloning plasmids and in conjugation experiments to generate *P. aeruginosa* chromosomal mutants.

A list of all *P. aeruginosa* chromosomal mutants constructed and used in this study is provided in the key resources table.

*E. coli* cultures were propagated in LB Miller broth medium or on LB Miller agar (LA) plates supplemented with 20 μg/mL tetracycline, 10 μg/mL gentamicin, or 50 μg/mL kanamycin depending on the resistance marker of the cloned plasmid.

For *P. aeruginosa* isolation or subculture on solid medium, Nutrient Agar (NA) medium, prepared with 40 g of Blood Agar Base and 5 g of Bacto Yeast extract per liter of medium, was used. Overnight or fresh liquid cultures of *P. aeruginosa* were grown on Nutrient Yeast Broth (NYB), prepared with 25 g of Nutrient Broth and 5 g of Bacto Yeast extract per liter of medium. All bacterial cultures were incubated at 37°C, with 220 rpm of shaking for liquid cultures, unless otherwise specified.

For all experiments, PAO1 strains were first streaked out onto fresh NA plates from a 40% glycerol stock stored at −80°C and grown overnight at 37°C. Overnight cultures were then inoculated from one single colony in 15 mL culture tubes filled with 3 mL of NYB. The following day, 1 mL of overnight cultures was centrifuged at 3500 RCF for 5 min, after which the supernatant was discarded and the pellet was resuspended in 1 mL of fresh NYB medium. Fresh cultures of 3 mL NYB in 15 mL culture tubes were inoculated by 1:100 dilution of the washed overnight suspension. OD600 measurements were done after 2–3 h of incubation and cultures were directly used for experimental setup.

### Method details

#### Construction of plasmids and *P. aeruginosa* PAO1 mutant strains

To construct chromosomal *P. aeruginosa* PAO1 mutants (deletion mutant or in-frame insertion of a fluorescent protein or Strep-tag), the suicide vectors pEXG2^44^ or pME3087^45^ were used. Briefly, regions of approximately 500 bp directly flanking the gene segment to be edited were amplified by PCR using the Q5 High-Fidelity DNA polymerase, alongside the tag or fluorescent protein of interest (when applicable). Details about primers used in this study and plasmid construction can be found in Tables S1 and S2, respectively. Plasmid cloning was done using the NEBuilder HiFi DNA assembly Master Mix according to the manufacturer’s instructions. Linearised plasmid backbones and inserts with compatible ends (generated by PCR using the Q5 High-Fidelity DNA polymerase) were used in the reaction. After transformation of the reaction products into chemically competent *E. coli* DH5α cells and plating onto selective LA medium, colonies were screened by colony PCR using GoTaq DNA polymerase. Correct clones were systematically checked by Sanger Sequencing (Microsynth) to confirm that the cloned plasmids had inserts with the expected sequences.

The cloned suicide vectors were subsequently introduced into *P. aeruginosa* PAO1 by triparental mating using the *E. coli* HB101 strain (pRK2013). Selection of the first recombination event was done by plating on selective NA medium (10 μg/mL chloramphenicol plus either 100 μg/mL tetracycline for pME3087 or 50 μg/mL gentamicin for pEXG2). A counter-selection was done by enrichment for tetracycline-sensitive clones (pME3087-conjugated) as previously published, doing a 4-h incubation with carbenicillin instead of D-cycloserine,^46^^,^^47^ or by streaking of the pEXG2-conjugated clones onto LA Lennox (prepared from LB Lennox and European Bacteriological Agar) supplemented with 5% sucrose (levansucrase counterselection). Clones were screened by colony PCR using GoTaq DNA polymerase and double-checked for loss of tetracycline (when using pME3087) or gentamicin resistance (when using pEXG2) by patching onto selective medium. Correct clones were restreaked to purity and overnight cultures were inoculated using a single colony. Strains were stored at −80°C after mixing a 1:1 volume of autoclaved 80% glycerol and overnight cultures into a cryogenic vial.

#### Microscopy imaging

For all microscopy experiments, bacteria were grown until OD_600nm_ = 0.3–0.8 in 3 mL of NYB medium. When needed, membrane staining with FM4-64 was done by adding 0.3 μL of 10 mM FM4-64 (in DMSO) to 100 μL of culture in an Eppendorf tube. An incubation was done at 37 °C for 10 min (no shaking) before proceeding with imaging. The cells were deposited onto an agarose pad containing 1% UltraPure Low Melting Point agarose dissolved in 0.5*X* phosphate-buffered saline (PBS).

Phase contrast and widefield fluorescence microscopy images were acquired on a Zeiss Axio Imager 2 equipped with an objective alpha Plan-Apochromat 100×/1,46 Oil Ph3 M27 and a ZEISS Axiocam 305 mono CCD camera.

Structured Illumination Microscopy (SIM) fluorescence images were acquired with the ELYRA7 microscope (Zeiss) using excitation lasers of 488 nm (for msfGFP), 561 nm (for mCherry or FM4-64) or 405 nm (for mTagBFP2). Laser intensities of 6–8% and exposure times of 60–80 ms were used for imaging of fluorescently-tagged proteins, while 0.5–1% laser was sufficient to visualise the FM4-64 membrane dye.

For time-lapse acquisition, cells were instead deposited onto an agarose pad containing 1% UltraPure Low Melting Point agarose dissolved in 0.25*X* LB broth. An acquisition interval of 5000 ms between each frame and a total of 25 cycles were chosen. The software definite focus function was used to prevent loss of focus over time. Reconstruction was done using the Processing tab of the Zen software (SIM^2^ algorithm) using the parameters shown in Figure S10.

#### Image processing

All image treatments were performed using Fiji.^22^ Representative microscopy images and images to be analyzed were opened in Fiji and cropped when needed to keep only cells showing good in-focus signal. Background noise was measured in an area devoid of cells and the mean background signal value was subtracted from the entire image by using the Process -> Math -> Subtract function. When showing merged representative images, merging of the separated phase-contrast and fluorescence channels was done using the Image -> Color -> Merge Channels function. When a shift between the phase contrast channel and the fluorescence channel was observed (e.g., several foci or cell background signal being outside of phase contrast cells all in the same direction), this was manually corrected for the entire image using the Image -> Transform -> Translate function. Display was adjusted using the Reset function in the Adjust Brightness/Contrast menu for most images. For FISH images, the display range was manually set to be identical between images of the WT and the *rne*ΔMTS strain (and also with the negative control when possible).

#### Growth assays on solid media

For solid medium growth assays, square Petri dishes (12 × 12cm) were filled with 50 mL of warm NA. For testing growth with 0.75 M NaCl or KCl, 17.54 g of NaCl or 22.36 g of KCl were added to 400 mL of freshly prepared NA medium and autoclaved before pouring into square Petri dishes. For testing growth with 0.75 M sucrose, a 2*X* NA concentrate was prepared and mixed with an equal volume of 1.5 M sucrose solution when still hot before pouring into a square Petri dish. The Petri dishes were left to dry on the bench for 24h–36h before use.

For the spotting assay, fresh cultures (OD_600nm_ = 0.4–0.8) were adjusted to OD_600nm_ = 1 by centrifugation and serially diluted from 10^–1^ to 10^–7^ in a 96-well plate using a multichannel pipette. 5 μL of each dilution series were then spotted using a multichannel pipette onto pre-dried supplemented or standard NA plates. Plates were incubated upside down at 37 °C or 16 °C for approximately 24 h or 48 h, respectively.

For the screening shown in Figure S5 and 50 mL of warm NA medium was similarly supplemented with the indicated chemicals before pouring into a square Petri dish. Pretreatment conditions correspond to the exposure of an exponentially growing culture, adjusted to OD_600nm_ = 1 by centrifugation, to the indicated stress. For the ethanol stress, 99% ethanol was added to 250 μL of bacterial suspension and incubated at room temperature. For the heat stress, 100 μL of bacterial suspension was transferred into an Eppendorf tube and incubated for the indicated time in a water bath pre-heated to 53°C. Temperature was accurate, as verified with a thermometer. Cultures were then similarly diluted in 96-well plates and spotted onto pre-dried NA plates using a multichannel pipette.

#### *Galleria mellonella* killing assays

To conduct *G. mellonella* infection assays, fresh cultures of *P. aeruginosa* PAO1 WT, *rneΔMTS* or *rneMinDα* were adjusted to a OD_600nm_ = 1 and serially diluted to 10^–6^ in Dulbecco’s Phosphate-Buffered Saline (dPBS) in a 96-well plate using a multichannel pipette. Three 10 μL drops of each 10^–6^ dilution were spotted onto an NA plate to ensure that CFU counts were similar between the strains. Ten drops of 10 μL were placed onto a clean Parafilm sheet. Each entire 10 μL drop was injected using an Omnican 50 sterile insulin syringe into the second-to-last leg of one larva, facing toward the head. For every Petri dish containing ten larvae, the time of injection was recorded immediately after injection of the last larva, and the Petri dish was placed into a dark incubator at 37°C. Of note, the maximal time difference between injection of the first and the last larvae per Petri dish is 12 min. A control group of larvae was also included, in which each larva was similarly injected with 10 μL of sterile dPBS instead. The following day, deaths were monitored every hour. A larva was considered dead if it completely stopped responding to stimuli (touched on the legs or the head with a pipette tip). Time of death was calculated as the interval of time between the recorded time of injection and death occurrence. Survival curves were plotted and analyzed with GraphPad Prism. Three independent biological replicates (20–30 larvae per replicate and per strain) were conducted in which all three strains tested were subcultured, diluted, and injected into the *G. mellonella* larvae in parallel, together with the mock dPBS injections.

#### RNA-seq sample collection

The *rne*ΔMTS strain was grown and harvested in parallel to the WT strain whose data are already published in Geslain et al.^20^ The analysis was thus done comparing the *rneΔMTS* dataset to the RNA-seq dataset of PAO1 WT from Geslain et al.^20^ To obtain three biological replicates, three independent cultures of PAO1 WT and *rneΔMTS* (inoculated from three different overnight cultures originating from three distinct streaks on solid medium) were grown on the same day in parallel at 37°C with shaking in 50 mL Erlenmeyer flasks containing 10 mL of NYB until OD_600nm_ = 1.0–1.2. 500 μL of each culture were immediately transferred into separate 1.5 mL tubes pre-filled with 1 mL of RNAlater. After harvesting was completed for all replicate cultures, centrifugation of the tubes was done for 30 min at 9600 x *g*. The supernatant was discarded and pellets were stored at −80°C.

For bacterial lysis, two pellets for each strain and replicate culture were resuspended and pooled in a total of 250 μL of 1*X* Tris-EDTA (TE) buffer supplemented with 1 mg/mL of lysozyme. After a 5-min incubation at room temperature, total RNA was extracted using the Monarch Total RNA Miniprep kit following the manufacturer’s instructions. Following RNA purification, a final DNA removal treatment was done using TURBO DNase according to the manufacturer’s instructions. gDNA-free total RNA was purified using the RNA purification columns from the Monarch Total RNA Miniprep kit, following the priming, washing and elution steps recommended by the manufacturer. The absence of residual gDNA was confirmed by doing a 35-cycle PCR using a *rpoD*-targeting primer pair. Quality check, ribosomal RNA depletion and RNA sequencing were conducted by Novogene.

#### GC-EMOTE library construction

The GC-EMOTE experiment was performed starting from the same RNA samples used for RNA sequencing. The protocol published by Redder (2018) as used as a template and optimised for GC-rich organisms like *P. aeruginosa* as follows.^29^ The primers and oligos used for the GC-EMOTE experiment are listed in Table S3.

To begin with, TURBO DNase-treated gDNA-free total RNA samples were depleted for rRNA using the riboPOOL kit for *P. aeruginosa*, following the manufacturer’s instructions and using 5 μg of total RNA as starting material. After rRNA depletion, the RNA samples were purified using the sodium acetate/ethanol precipitation method, using 1 μL of GlycoBlue Coprecipitant per sample instead of the linear acrylamide provided in the kit to improve pellet visualisation. After all the washing steps, drying of the RNA pellet was done for 5 min under a clean sterile flow hood and the pellet was resuspended in 10 μL of nuclease-free 0.1*X* TE buffer. For ligation of the BioVm1 RNA oligo, a ligation Master mix was prepared in the following proportions (per sample): 2 μL 10*X* T4 RNA ligase 1 buffer, 2 μL of 10 mM ATP, 0.8 μL RNasin Plus Ribonuclease Inhibitor, 4.2 μL PEG8000 50%, and 1 μL T4 RNA ligase 1. A mixture of 9 μL of rRNA-depleted RNA sample plus 1 μL of 100 μM BioVm1 was prepared, heated to 80 °C for 2 min and flash-cooled on ice. The ligation mixture was then added to the samples and incubated for >16 h at 16 °C. Ethanol/sodium acetate precipitation was then done as described by Redder^29^ with minor changes: two volumes of ethanol were added instead of three and washing was done with 400 μL of cold 70% ethanol. Final resuspension of the pellet, after 5 min drying under the sterile flow hood, was done in 10 μL nuclease-free 0.1*X* TE buffer.

For the reverse-transcription reaction, the ImProm-II Reverse Transcriptase was used. The Master Mix was prepared according to the manufacturer’s instructions and 1 μL of 20 μM DROCC primer was mixed with 9 μL ligated RNA and heated for 2 min at 80 °C before allowing a slow cooling to room temperature (5 min with tubes lying on top of the turned off heat block and 5 min in an Eppendorf rack). 10 μL of Reverse Transcription mix were added to each sample of RNA mixed with DROCC primer. Tubes were left at room temperature for 10 min then incubated at 42 °C for 60 min before heat-inactivating the reverse transcriptase at 70 °C for 15 min. The cDNA was purified with AMPure XP beads using a 1.5*X* ratio of beads to sample and following the manufacturer’s instructions. Elution was done in 40 μL of nuclease-free 0.1*X* TE buffer.

For PCR amplification and tagging, a Mastermix was prepared as follows (per sample): 7.5 μL of UltraPure DNase/RNase-free Distilled Water, 10 μL of 5 M betaine, 1.5 μL of DMSO, 10 μL of 5*X* Q5 reaction buffer, 4 μL of 2.5 mM dNTPs, 2.5 μL of 10 μM primer E2, and 0.5 μL of Q5 High-Fidelity DNA polymerase. After splitting 36 μL of MasterMix into different PCR tubes, 10 μL of purified cDNA sample and 2.5 μL of 100 nM primer pVmA, pVmB, pVmC, pVmD, pVmE, pVmF, pVmG, pVmH, pVmI, pVmJ, pVmK, or pVmL (one unique primer for each cDNA sample) were added per tube to allow the generation of a unique tag for each sample. A first short touchdown PCR was run with the following program: 98 °C for 2 min; 5 cycles of 98 °C for 10 s, 67 °C (1x)/66 °C (1x)/65 °C (1x)/64 °C (1x)/63 °C (1x) for 30 s, 72 °C for 45 s. At the end of the program, PCR tubes were quickly placed on ice, supplemented with 2.5 μL of 10 μM primer E1, and run with a second touchdown PCR program: 98 °C for 1 min 50 s; 25 cycles of 98 °C for 10 s, 67 °C (1x)/66 °C (1x)/65 °C (1x)/64 °C (1x)/63 °C (21x) for 30 s, 72 °C for 1 min; 72 °C for 5 min. At the end of the run, the barcoded PCR products were pooled per series of replicates and purified with AMPure XP beads using a 0.8*X* ratio of beads to sample according to the manufacturer’s instructions. At the end of the purification, the pooled dsDNA library was eluted in 50 μL mQH_2_0 and stored at −20 °C. Sequencing was done on the Novaseq X Plus Series (PE150) platform at Novogene (120 G raw data per sample).

#### RNA-seq processing and mapping

Reads were mapped using Bowtie2^48^ to *Pseudomonas aeruginosa* PAO1 genome assembly ASM676v1 and counts were aggregated using summarize Overlaps in the package GenomicAlignments^49^ in R^43^ using the ASM676v1 gtf version 2.2 (NCBI accession: GCF_000006765.1). Differential expression was performed using DESeq2^50^ and transcripts were considered differentially expressed between two conditions if the absolute value of log2FC between conditions was greater than 1 (i.e., 2-fold up or down) with an adjusted *p*-value less than 0.05. To find functional enrichment among differentially expressed gene groups, Gene Ontology (GO) enrichment was assessed using adjusted *p*-values from hypergeometric tests. The number of reads covering each genomic position in each replicate of each strain was calculated to give RNASeq depth. This is normalised by the total number of reads in each sample and later compared with the normalised number of 5′ EMOTE ends at the same locus, allowing us to see local changes in transcript depth around cleavage sites between strains and compare cleavage events to the relative number of potential transcripts available to cleave.

#### GC-EMOTE sequencing data processing and mapping

Raw sequencing data were demultiplexed by barcode using custom Bash scripts, trimming the four nucleotide barcodes from the 5′ end as they were identified, along with the single random nucleotide upstream. Adapter trimming was then performed on the remaining downstream read using cutadapt v4.9.^51^ A 19-nucleotide overlap with zero error rate was required, and the 5′ fixed tag sequence CTTCACCACACCCTATCAC was removed from reads. Only trimmed reads were retained for downstream analysis. Unique molecular identifiers (UMIs) were extracted from the 5′ ends of reads using UMI-tools v1.1.6 with a regular expression capturing the 17-nucleotide UMI (excluding guanine) immediately upstream of the fixed ACATA motif.^52^ Reads were further length-trimmed to 25 nucleotides using cutadapt for consistent downstream alignment.

Trimmed reads were aligned to the *Pseudomonas aeruginosa* PAO1 genome assembly ASM676v1 using bowtie2 v2.5.4 in end-to-end, very-sensitive mode.^48^ This mode configures short 20-bp seed lengths (-L 20), strict zero-mismatch seed anchoring (-N 0), and a dense seed interval function (-i S,1,0.50). To recover transcript boundaries spanning single nucleotide variants or repetitive regions without soft-clipping, search parameters were maximized to allow 20 consecutive seed-extension trials (-D 20) and up to 3 separate reseeding attempts (-R 3). SAM alignments were converted to BAM format, sorted, and indexed with samtools v1.20.^53^ PCR duplicates were removed using UMI-tools with the directional method, leveraging UMI sequences to identify and collapse duplicate reads.

Five prime end depths were calculated genome-wide (the number of strand-specific 5′ ends of reads at each position) and quantified relative to transcript features using ASM676v1 gtf version 2.2 (NCBI accession: GCF_000006765.1) for locations. In EMOTE analysis, the start of the reads represents the exact positions where RNA molecules have been enzymatically cleaved and the 5′ end depths are quantified because they pinpoint the precise single-nucleotide resolution of those cleavage sites across the genome. Each cleavage site has a dinucleotide sequence immediately downstream of the cut, the first two nucleotides at the 5′ end of the read. In order to predict whether dinucleotides present at cleavage site loci indicated by GC-EMOTE 5′ ends were compatible with RNase E cleavage, a score based on the RNase E consensus logo plot sequence published in Chao et al.^34^ was generated.

To predict whether dinucleotides flanking the cut-site loci identified by GC-EMOTE 5′ ends were compatible with RNase E cleavage, a quantitative sequence affinity scoring matrix was constructed from the published consensus logo plot.^34^ Positions +1 and +2 relative to the cleavage site were selected for scoring because they exhibited the highest sequence bias (information content) across the recognition motif. The raw base frequencies (x) for Adenine, Uracil, Cytosine, and Guanine at these two positions were extracted from the logo plot and converted to a linear weight scale using an antilog transformation (2^*log2(x)*^). Because positions +1 and +2 interact independently with the enzyme, the joint probability of any given dinucleotide sequence combination was calculated by multiplying their individual position weights (*p*1 ∗ *p*2). To account for the mathematical properties of the raw plot extractions where the position vectors did not sum to unity (*R*1 = *sum*(*p*1); *R*2 = *sum*(*p*2)), a normalization constant (*scale*_*factor* = *R*1 ∗ *R*2) was applied to the joint probability calculation:

$$Weight={\mathrm{scale}}_{\mathrm{factor}}\;*\;p_{1}\left({\mathrm{base}}_{1}\right)\;*\;p_{2}\left({\mathrm{base}}_{2}\right)$$

This computational approach translates visual logo frequencies into a digitized, 16-row look-up matrix ranging from highly preferred targets (e.g., AU and UU) to strongly avoided sequences (e.g., GG). By applying these weights to the genome-wide data, true enzymatic processing events can be robustly differentiated from random background RNA degradation and sequencing depth biases.

To give an example, if +1 bits are [A = 0.22, U = 0.13, C = 0.06, G = 0.02] and +2 bits are [U = 0.80, C = 0.12, A = 0.05, G = 0.04], these compressed logarithmic values are first converted to a linear weight scale using an antilog transformation (2^∧^bits). Adding these individual linear weights together establishes a total cumulative pool size of 4.32 for position +1 (R1) and 4.89 for position +2 (R2). Multiplying these two position totals together yields a baseline normalization constant (scale_factor = R1 ∗ R2) of 21.11. The final compatibility score for any given sequence combination is then determined by multiplying this scale factor by the independent position weights, as above.

For an AU dinucleotide, the most common dinucleotide downstream of an RNase E cleavage site, the calculation multiplies the scale factor (21.11) by the individual transformed weights of Adenine at position +1 (2^∧^0.22 = 1.16) and Uracil at position +2 (2^∧^0.80 = 1.74) to yield a final prioritized score of 42.8.

TMH locations for the entire transcriptome were predicted using DeepTMHMM 1.0.24, with default parameters.^54^

#### Single molecule fluorescence *in situ* hybridization (smFISH)

TAMRA-labelled ssDNA probes for FISH were synthesised by the Stellaris RNA FISH probes service (Biosearch Technologies). A set of 47–48 probes was used to target putP, asrA, or pqsAB mRNAs (Table S4). Of note, pqsAB was chosen instead of pqsA alone due to the difficulty of getting a sufficient number of specific probes binding in pqsA only. The Stellaris FISH probe designer was used as a base generator of potential FISH probes (18-, 19- or 20-mers), after which all the candidate probes were aligned against the *P. aeruginosa* genome (NC_002516) using Snapgene. Probes with poor specificity (approx. > 20–30 potential binding sites) or with unspecific sense strand targets having a predicted Tm of >50 °C were typically excluded from the dataset. A minimal spacing of two nucleotides between each probe was chosen, with a preferential selection of probes allowing a spacing or three or more. Finally, the probe length was manually adjusted to achieve similar Tm values for most probes based on PerlPrimer calculations.^42^

The smFISH protocol from Skinner et al.^37^ was used as a base protocol. All reagents were as described in the published protocol unless otherwise specified.^37^ A few changes were made to the protocol, as described hereafter.

Fresh cultures were started in 10 mL NYB (in 100 mL Erlenmeyer flasks) for asrA/pqsAB, or in 15 mL NYB (in 250 mL Erlenmeyer flasks) for putP. 1 mL of each overnight culture was centrifuged, resuspended in NYB, and diluted 1000-fold for inoculation of the fresh cultures. The cultures were cross-linked when OD_600nm_ was ≈0.4 for FISH against putP or ≈1 for FISH against pqsAB/asrA. A 5*X* fixing solution containing a 1:1 mix of formaldehyde 36.5–38% and 1*X* RNase-free PBS was directly added (1/4 volume) to the cultures for cross-linking. The Erlenmeyer flasks were sealed with parafilm and incubated for 15 min at 37 °C with shaking, followed by a 30-min static incubation on ice. A volume of culture corresponding to *V* (mL) = 4/OD_600nm_ x 1.2 was then transferred into 50 mL Falcon tubes and centrifuged at 800g with slow braking, 4 °C, for 20–25 min. The subsequent washing and permeabilization steps were performed as described by Skinner et al.,^37^ increasing the centrifugation times from 3.5 min to 6 min. The hybridisation solution described by Skinner et al.^37^ and containing formamide, dextran sulfate, SSC buffer, tRNA, Ribonucleoside Vanadyl Complex (RVC), and Ultrapure BSA, was altered as follows: the concentration of RVC was increased from 2 mM to 10 mM and tRNA from *S. cerevisiae* was used instead of *E. coli-*derived tRNA. FISH probes were briefly denatured by heating at 80 °C for 3 min and placing them on ice before adding them to the hybridisation mix to a final concentration of 1 μM. After resuspension of the permeabilised cells into the hybridisation mix, the suspension was heated at ≈ 67 °C for 5 min in a heat-block, plus an additional 10 min after turning off the heat block. A slow 5-min cool-down was then done by leaving the tubes lying on top of the heat block before placing them in an Eppendorf shaker at 30 °C overnight (450 rpm shaking). Of note, FISH probes and suspensions containing FISH probes were shielded from light at all times.

The next day, washing steps were performed mostly as described by Skinner et al.,^37^ reducing the incubation times to 20 min and performing an additional final 10-min wash in 2*X* SSC buffer before putting ≈ 1–2 μL of suspension onto a pad made with 0.5*X* RNase-free PBS and 1% TopVision Low Melting Point Agarose. Visualisation was done using epifluorescence microscopy (800 ms exposure time for the TAMRA channel).

#### RNase E foci or smFISH foci quantification/subcellular localisation analysis

All image treatments and analysis were performed using Fiji.^22^ Images to be analyzed were first opened in Fiji and processed as described in the Image Processing section.

Detection of local maxima and their assignment to the detected bacterial particle was done with MicrobeJ.^21^ Subsequent quantification of foci counts per cell, foci intensities (or other characteristics) and generation of average local maxima subcellular localisation were also generated with the MicrobeJ plugin.^21^ Parameters used for bacteria and maxima detection of images showing either msfGFP-tagged RNase E variants or mRNAs visualised by FISH are shown in Figure S11. Manual segmentation of cells was done in some cases to avoid excluding many cells from the analysis, always manually verifying that the foci count has not been affected for segmented cells. Erroneous automatic detection of cells was manually corrected by removing those elements from the results table. The results were then exported as a.csv file. Data obtained for several individual images acquired from three independent biological replicates was pooled. Graphs of foci intensity and other foci morphology measurements were then plotted using GraphPad Prism. Graphs showing the average foci subcellular localisation (RNase E foci or FISH foci) were generated directly with MicrobeJ.^21^ The “XYCellDensity” graphing method was selected, and the density bandwith settings used were x = 0.05 and y = 0.25 for all graphs generated. The default look-up table (“fire”) was chosen for all graphs. Images were saved as.svg files and colors were inverted in Adobe Illustrator with the Edit Colors -> Invert Colors function.

#### Swimming and twitching assays

Swimming assays were conducted using square Petri dishes (12 × 12cm) filled with 50 mL of a medium containing tryptone (10 g/L), sodium chloride (5 g/L) and European Bacteriological Agar (3 g/L). The plates were dried for approximately 10 min under the flow hood before inoculating them by injecting 1 μL of exponentially growing PAO1 cultures inside of the agar layer.

Twitching assays were done using LB Lennox broth supplemented with European Bacteriological Agar (10 g/L). Square Petri dishes (12 × 12cm) were filled with 25 mL of autoclaved medium and inoculated by picking an isolated colony of the PAO1 strain of interest and applying it to the bottom of the plate by stabbing through the agar and keeping the tip firmly pressed down for 5 s.

Swimming and twitching assay plates were incubated at 37°C for 24 or 48 h before imaging. To visualize twitching plates, the agar layer was carefully peeled off and a 0.1% crystal violet solution was applied onto the plate for a few minutes before rinsing three times with water. After image acquisition, quantification of the area of swimming or twitching was performed using Fiji.^22^

#### Western Blot

Western Blots were performed following the protocol described in Geslain et al.^20^ Briefly, exponential cultures of PAO1 chromosomally expressing RNase E-Strep, RNase E^ΔMTS^-Strep or RNase E^MinDα^-Strep were harvested in equal amounts and resuspended in an identical volume of 1x Laemmli buffer before heating samples for 10 min at 95°C. After running the samples onto a polyacrylamide gel, transfer onto a nitrocellulose blotting membrane was performed. Following overnight blocking at 4°C, incubation with the primary antibody (StrepMAB-Classic Monoclonal antibody or *Pseudomonas aeruginosa* oprF polyclonal antibody) diluted in PBS-Tween 0.1% + 5% milk was done for 2 h. After three washing steps with PBS-Tween 0.1%, incubation with the secondary antibody (Anti-mouse or Anti-rabbit IgG-peroxidase antibodies) diluted in PBS-Tween 0.1% + 5% milk was done for 1 h, after which an additional three washes with PBS-Tween 0.1% were performed. A final wash with PBS was performed, then signal was revealed with SuperSignal West Pico PLUS Chemiluminescent Substrate and visualized with ChemiDoc MP Imaging System (Bio-Rad).

#### RTqPCR

RT-qPCR verification of relative transcript abundance in the WT or *rne*ΔMTS RNA samples was done following the protocol from Geslain et al.^20^ The genes *rpoD*, *putP*, *asrA*, *pqsA*, *mexX* and *kdpA* were targeted using the primer pairs q65-q66, q116-q117, q120-q121, q112-q113, q118-q119, and q114-q115, respectively. Primer sequences are disclosed in Table S1. Briefly, a 500 ng aliquot of total gDNA-free RNA samples used in RNA-seq was converted to cDNA using the Improm-II Reverse Transcriptase. qPCR reactions were prepared after cDNA dilution using the SensiFAST SYBR Hi-ROX kit. Running conditions were: 95°C for 2 min, 39 cycles of 95°C for 5s, 60°C for 20s, 72°C for 15s, melt curve 65°C–95°C increment 0.5°C.

To calculate the fold-change enrichment of the tested transcripts in the *rne*ΔMTS relative to the WT sample, the 2^−ΔΔCt^ method was used.^55^

### Quantification and statistical analysis

Statistical analyses were performed using either R^43^ or GraphPad Prism. Statistical analyses of foci counts and morphological parameters (Figures 1B and 6D) were done using unpaired T-tests with Welch’s correction using GraphPad Prism 10. A normal distribution of the data was assumed. *n* values are indicated on the figures and correspond to the number of cells (for measurements of cell length or foci counts per cell) or to the number of detected foci.

To evaluate differences in the proportions of foci with distinct subcellular localisation (Figure 1C) or counts per cell (Figure 6C) across bacterial strains, a two-sample Chi-square test for equality of proportions with continuity correction was done using the R statistical software. *n* values are indicated on the figures and correspond to the number of detected foci (Figure 1C) or to the number of cells (Figure 6C). The same statistical test was also used to assess differences in the proportions of transcripts encoding proteins with distinct localisation signals (Figure 5D). *n* values for overall genome proportions were retrieved from Lewenza et al.^28^ *n* values within the group of downregulated transcripts (*n* = 39) are as follows: Type I: 4; Type II: 0; Type IV: 0; TAT: 1; TMH: 4; None: 30. *n* values within the group of upregulated transcripts (*n* = 54) are as follows: Type I: 10; Type II: 4; Type IV: 0; TAT: 0; TMH: 23; None: 16.

Quantification of significant differences in the data groups shown in Figures 5H and 5I was performed using the statistical software R^43^. A pairwise two-sided Welch’s *t* test (Figure 5H) and a two-sided Fisher’s exact test (Figure 5I) were performed. *n* values are indicated on Figure 5I and are also applicable to Figure 5H (Group A: 234; Group B: 271, Group C: 154; Group D: 165).

To assess differences in *G. mellonella* killing by different PAO1 strains (Figure 4B), a Kaplan-Meier survival analysis with a log rank (Mantel-Cox) test was performed using GraphPad Prism 10. *n* values and hazard ratios are indicated in the figure legend.

To report statistical significance, a 99% confidence interval was chosen for all tests described above except for the two-sided Fisher’s exact tests (95% confidence interval). All *p*-values are indicated in figure legends.

## Supplemental information

Document S1. Figures S1–S11 and Tables S1–S4

Video S1. SIM time-lapse of RNase E-msfGFP (green) in cells stained with FM4-64 (magenta)

Video S2. SIM time-lapse of RNase EΔMTS-msfGFP (green) in cells stained with FM4-64 (magenta)

Video S3. SIM time-lapse of RNase EMinDα-msfGFP (green) in cells stained with FM4-64 (magenta)
