# Supplementary material for: Decoding how RNase E membrane targeting controls RNA degradosome activity in Pseudomonas aeruginosa
Source: iScience. 2026 Oct 1;29(10):117717. doi: 10.1016/j.isci.2026.117717 (PMC13657362; doi:10.1016/j.isci.2026.117717)
Supplement: Supplementary file 1 [file mmc1.pdf]

iScience, Volume *29*

Supplemental information

Decoding how RNase E membrane targeting controls RNA degradosome activity in *Pseudomonas aeruginosa*

Sandra Amandine Marie Geslain, George Edward Allen, Johan Geiser, Peter Redder, and Martina Valentini

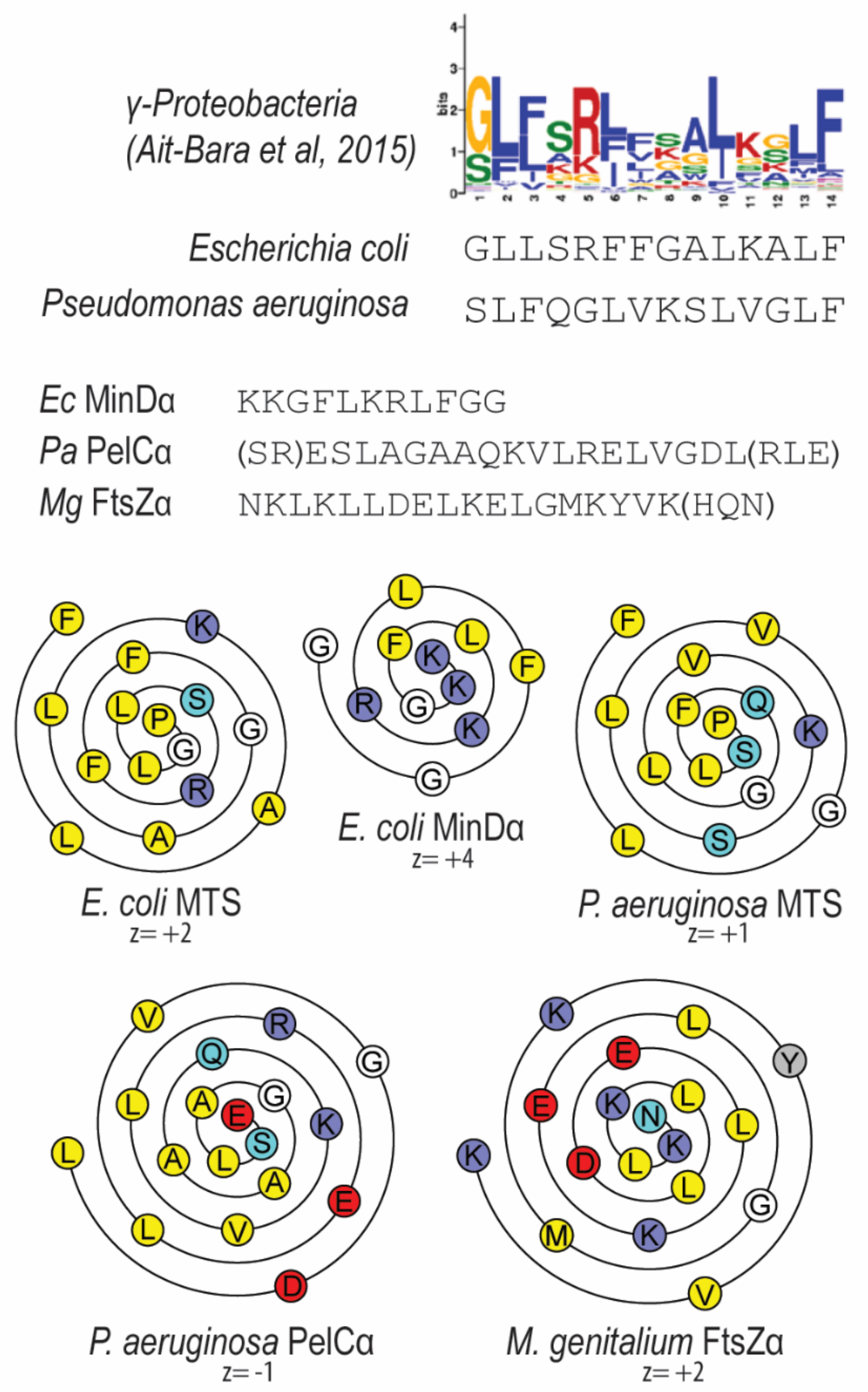

Figure S1: Amino acid sequence and helical wheel projection of the RNase E amphipathic helix (MTS) from *E. coli* or *P. aeruginosa*. A comparison to three unrelated amphipathic helices from the well-characterised *E. coli* MinD (MinDα)[1,2], the *P. aeruginosa* PelC (PelCα)[3], or the *Mycoplasma genitalium* FtsZ (FtsZα)[4] is shown. The RNase E MTS conservation logo plot from Ait-Bara et al.[5] is included for reference. Full indicated sequences of MinDα, PelCα and FtsZα correspond to the sequences used for construction of RNase E chimeras shown in Figure 1 and Figure S3. The amino acid indicated within brackets are not predicted to be part of the amphipathic helices. Amino acids were coloured based on their physicochemical properties. Yellow: hydrophobic; cyan: polar; blue: positively charged; red: negatively charged; white: glycine (neutral); grey: tyrosine (intermediate properties). Net charge (z) for each helix, calculated using HeliQuest[6], is indicated.

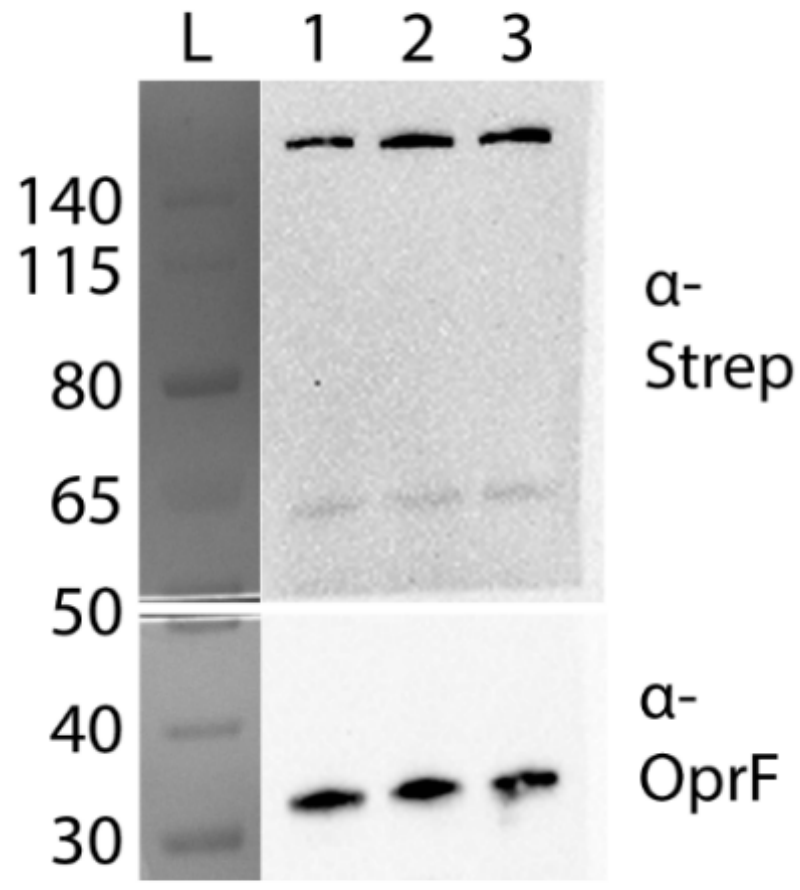

Figure S2: Western Blot of crude lysates of strains expressing RNase E-Strep, RNase $E^{\Delta MTS}$-Strep, or RNase $E^{MinD\alpha}$-Strep (chromosomal expression from the native locus). Protein levels are unaffected by the deletion of the MTS or the replacement of the MTS by the MinD amphipathic helix. 1: RNase E-Strep; 2: RNase $E^{\Delta MTS}$-Strep; 3: RNase $E^{MinD\alpha}$-Strep. Western Blot against the constitutive OprF protein was performed as a loading control.

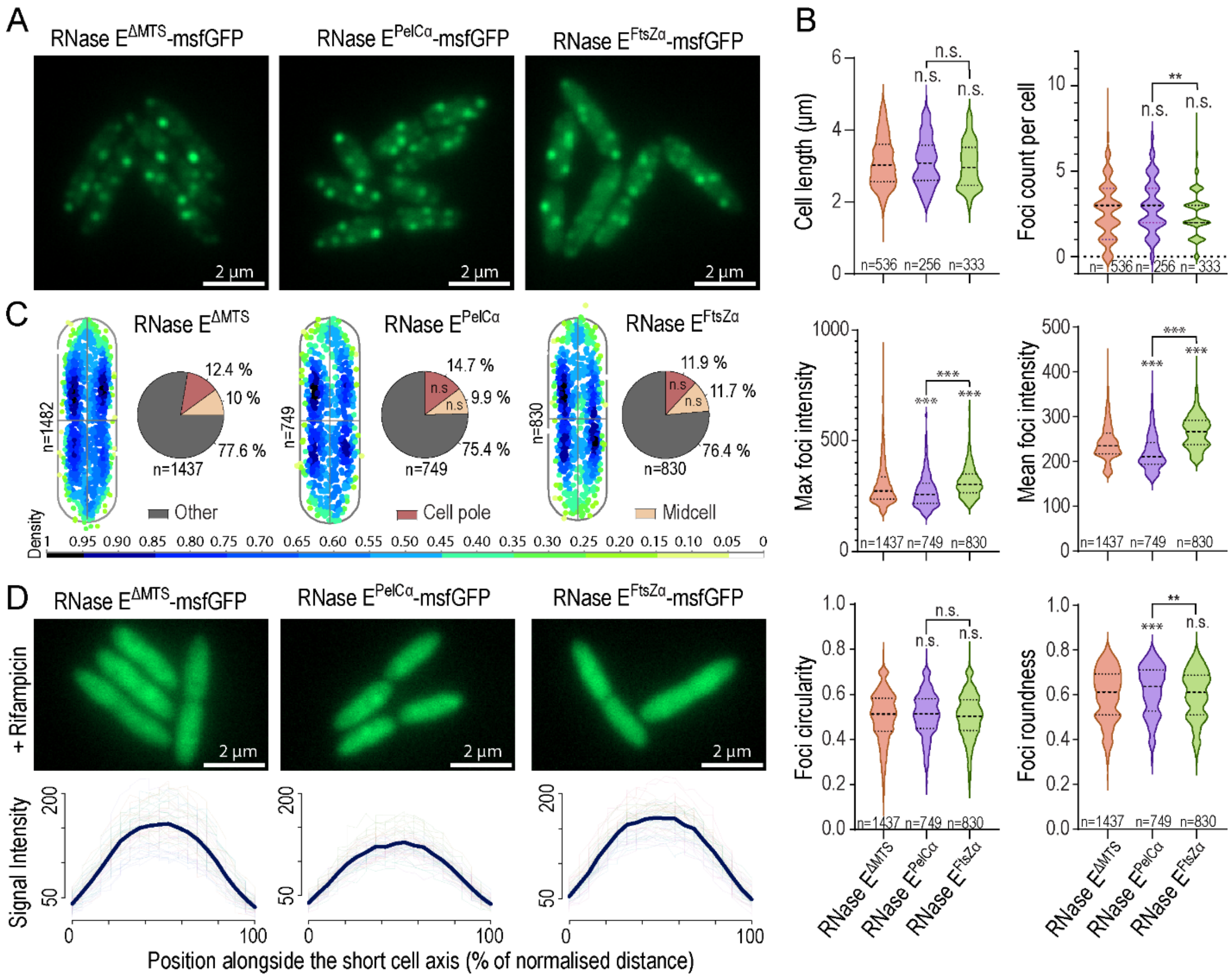

Figure S3: Reinsertion of the *P. aeruginosa* PelC or *M. genitalium* FtsZ amphipathic helices does not complement the foci morphology and localisation defects seen in an *rneΔMTS::msfGFP* mutant. (A) Representative images of RNase $E^{\Delta MTS}$, RNase $E^{PelC\alpha}$ or RNase $E^{FtsZ\alpha}$ C-terminally tagged with msfGFP (chromosomal expression from the native locus). **(B)** Violin plots (with median and quartiles) showing the quantification of cell length, RNase E foci count per cell, foci mean or max intensity and foci circularity or roundness from images of msfGFP-tagged RNase E variants (as described) acquired in three independent biological replicates. The analyses were performed using the MicrobeJ plugin of Fiji as described in Materials and Methods. An unpaired t-test with Welch's correction was done using GraphPad to compare mutant RNase E variants to the RNase $E^{\Delta MTS}$ (default comparison) or to each other (highlighted by brackets). n.s.: not significant (99% confidence interval); **: p-val < 0.001 ; ***: p-val < 0.0001. **(C)** XY cell density graph of the average distribution of RNase E mutants foci in a bacterial cell (left) and pie charts (right) highlighting the proportions of RNase E foci found at the midcell, the cell poles, or elsewhere ("Other"). Two-sample χ^2 tests were done using GraphPad and comparing the observed proportions in each chimeric RNase E relative to the RNase $E^{\Delta MTS}$. n.s.: not significant (95% confidence interval). Localisation of the foci was automatically detected using the MicrobeJ plugin. The XY cell density plots were generated using MicrobeJ whereby each detected foci, represented by a dot, is coloured based on its vicinity with neighbouring foci. For details about the settings used, see Materials and Methods. **(D)** Representative images (top panels) of RNase $E^{\Delta MTS}$, RNase $E^{PelC\alpha}$ or RNase $E^{FtsZ\alpha}$ C-terminally tagged with msfGFP (chromosomal expression from the native locus) after 30 minutes exposure to Rifampicin (100 µg/mL) and average fluorescence intensity profiles (bottom panels) from the short cell axis of the Rifampicin-exposed strains.

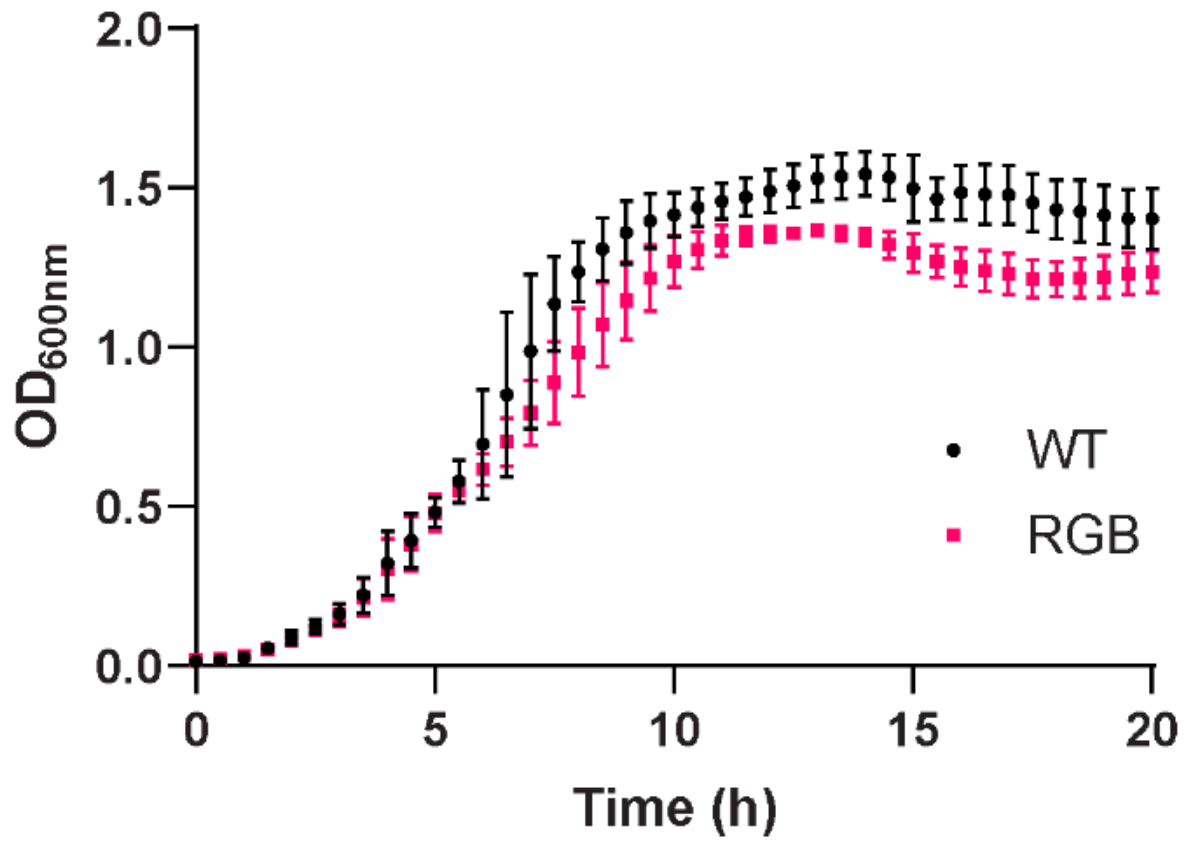

Figure S4: **PAO1 *rne::mCherry* / *rhl::msfGFP* / *pnp::mTagBFP2* ("RGB") grows similarly to the WT strain at 37°C in NYB broth** (96-well plate filled with 200 µL per well, inoculation at OD_{600nm} = 0.05).

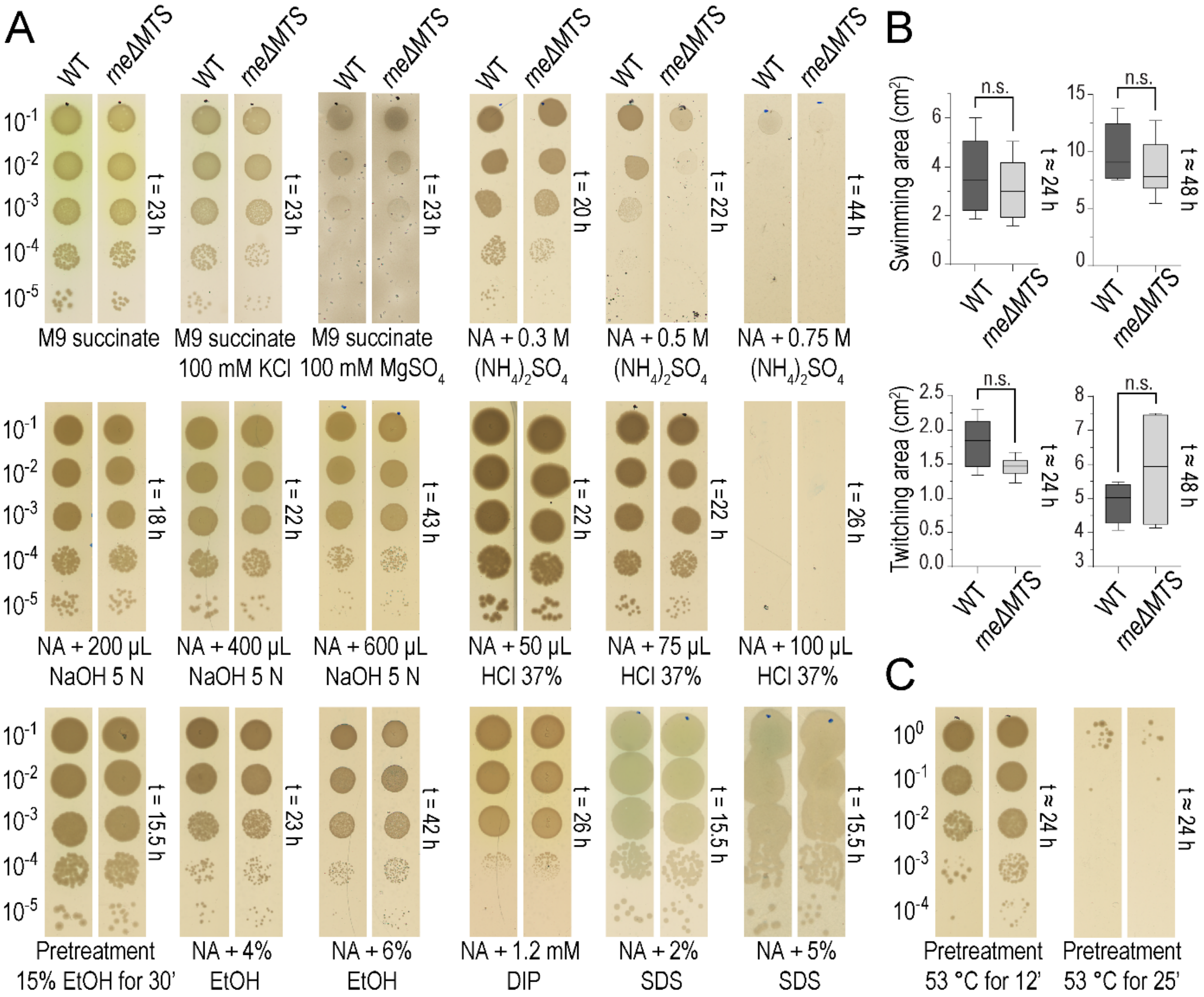

Figure S5: **Extensive phenotypic screening of the *rne*ΔMTS strain reveals a specific sensitivity to high salinity or acidic stress but no broad fitness cost.**

(A) Spot assay screening of *P. aeruginosa* WT or *rne*ΔMTS growth on solid media as indicated below each image. Time of imaging, recorded as hours post-inoculation, is indicated for each plate. Images shown are representative of one to two biological replicates. DIP: 2,2'-Bipyridyl; SDS: sodium dodecyl sulfate; EtOH: ethanol. **(B)** Swimming and twitching motility assays quantified after approximately 24h or 48h post-inoculation. Two to three independent biological replicates were performed and pooled for plotting. Statistical significance was assessed with a two-tailed unpaired t-test with Welch's correction. n.s.: not significant. **(C)** Spot assay on standard NA plates with the *P. aeruginosa* WT (left) or *rne*ΔMTS (right) strains after incubation of 100 µL of cultures in Eppendorf tubes in a 53 °C water bath for 12 or 25 minutes.

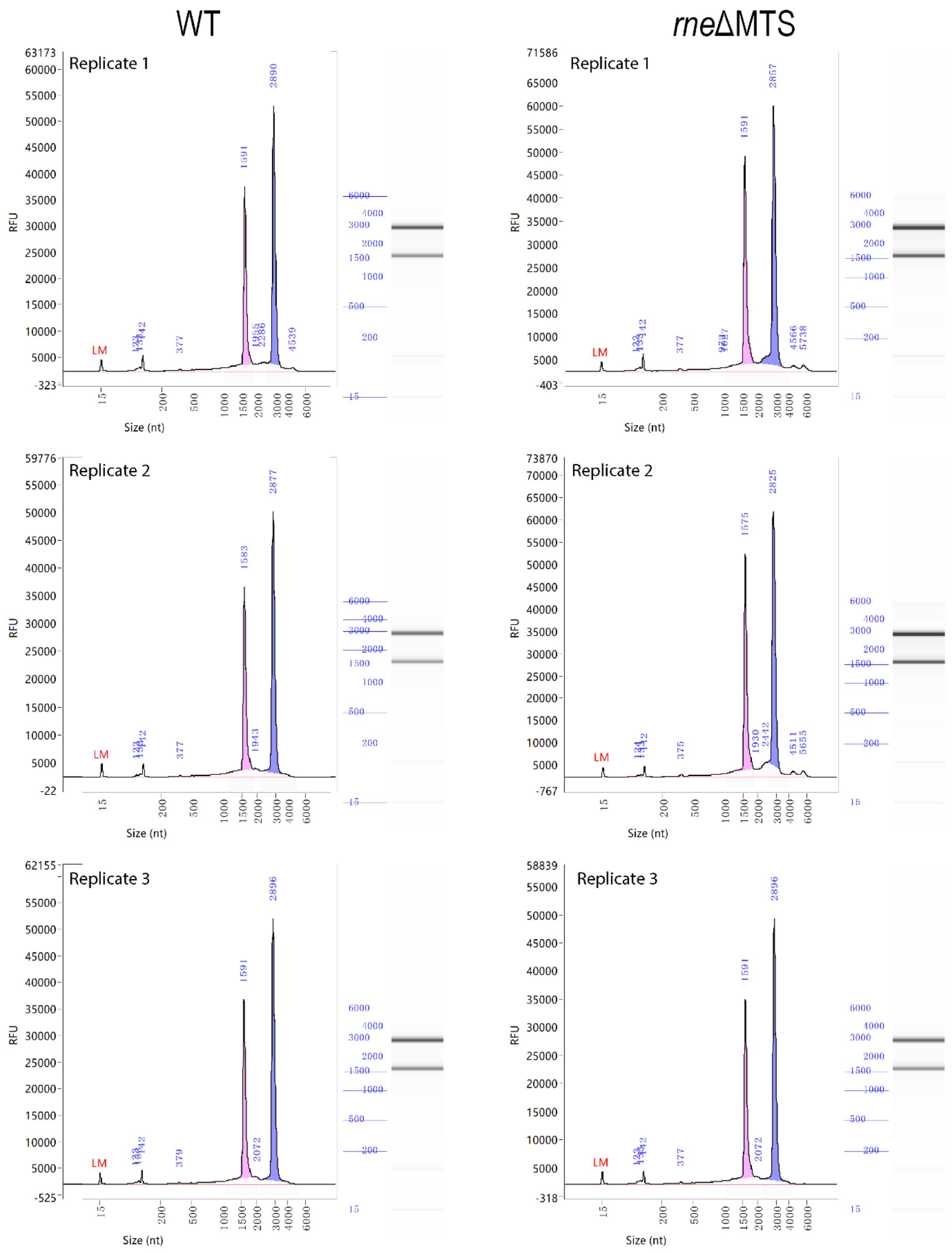

Figure S6: **Agilent 5400 peak profiles and simulated electrophoresis of total RNA samples of the WT or *rne*ΔMTS strains (biological triplicates).** The profile of each replicate is shown for each strain. Analysis was performed by Novogene prior to RNA sequencing.

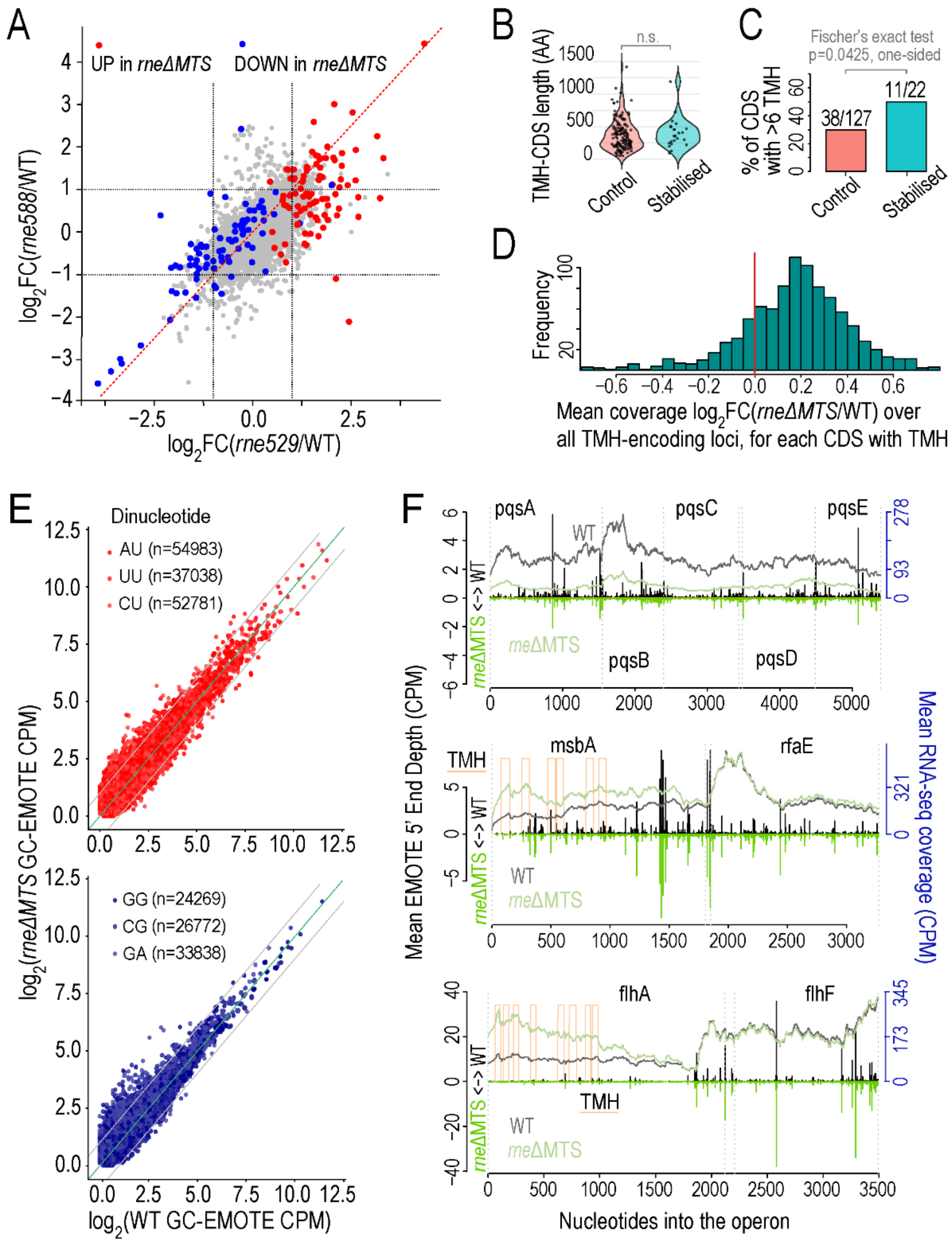

Figure S7: Additional analyses of RNA-seq and GC-EMOTE transcriptomics.

(A) Volcano plots highlighting genes commonly misregulated between the *rneΔMTS*, *rne529* and *rne588* mutant strains. **(B)** Comparison of CDS length in amino acids for transcripts encoding at least one TMH ("TMH-CDS") that are stabilised in *rne*ΔMTS versus WT (log2FC>1, adj. p-value<0.05, 'Stabilised') or unchanged (|log2FC|>1, raw p-value>0.1, "Control"). Lengths in the two groups were compared with a two-sided Welch's t-test. n.s.: not significant. **(C)** Comparison of the proportion of CDSs with more than 6 TMH in the Stabilised and Control groups detailed in (B). Proportions were compared with a one-sided Fisher's exact test. **(D)** Mean log2FC coverage ratio of *rne*ΔMTS / WT over all TMH-encoding loci, for each CDS encoding at least one TMH. TMH locations for the entire transcriptome were predicted using DeepTMHMM. **(E)** Scatter plots of GC-EMOTE unique monophosphorylated 5' ends in the WT strain (x axis, counts per million) or *rne*ΔMTS strain (y axis, counts per million) bearing either the most RNase E-compatible AU, UU, or CU dinucleotides (red graph, top) or the most RNase E-incompatible GG, CG, or GA dinucleotides (blue graph, bottom) at the

cleavage site position. Dinucleotide compatibility with RNase E cleavage was assessed by generating a score based on the RNase E consensus logo plot sequence published by Chao et al.[7]. Nucleotide weights (2^bits) for positions one and two were calculated from their graph of information content (bits) and multiplied to give the dinucleotide score. **(F)** GC-EMOTE peaks and RNA-seq coverage of an operon that loses coverage in *rne*ΔMTS (top panel) and two operons with TMH in the first CDS (bottom 2 panels). In these latter, the CDS with TMH are stabilised in *rne*ΔMTS, whereas those without TMH are not.

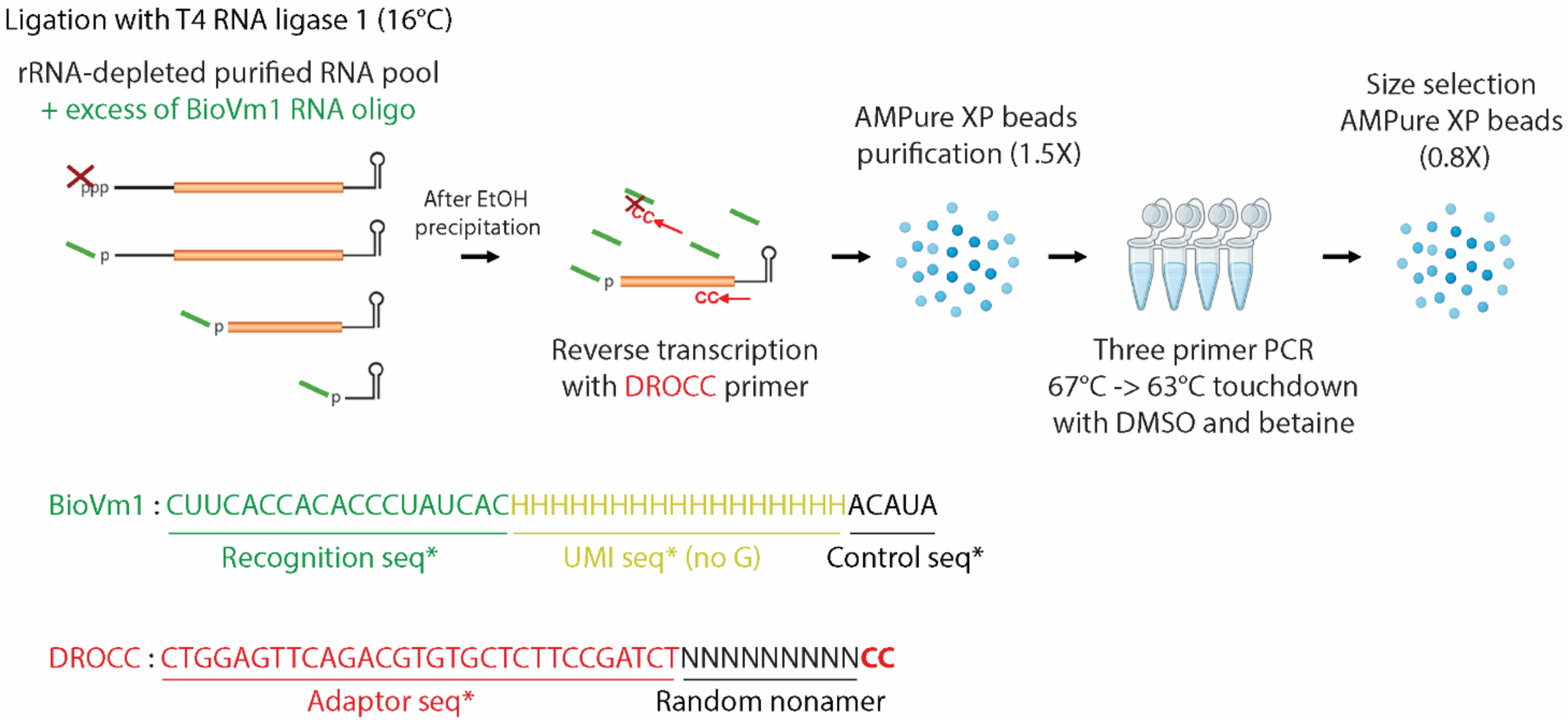

Figure S8: Experimental workflow for GC-EMOTE library construction. The major steps of the protocol are shown for clarity. Experimental details can be found in STAR Methods. The sequence of the BioVm1 RNA oligo (used in the ligation step) and the DROCC DNA oligo (used in the reverse transcription step) is shown for reference. The DROCC primer contains the fixed CC dinucleotide after a random nonamer sequence to avoid any potential annealing within the BioVm1 sequence. Initiation of reverse transcription can thus only occur at a position containing a GG dinucleotide within the target RNA molecule. Seq*: sequence. EtOH: ethanol. UMI: Unique Molecular Identifier (used to avoid PCR amplification biases). For an in-depth understanding about the logic behind the protocol, see Redder (2018)[8].

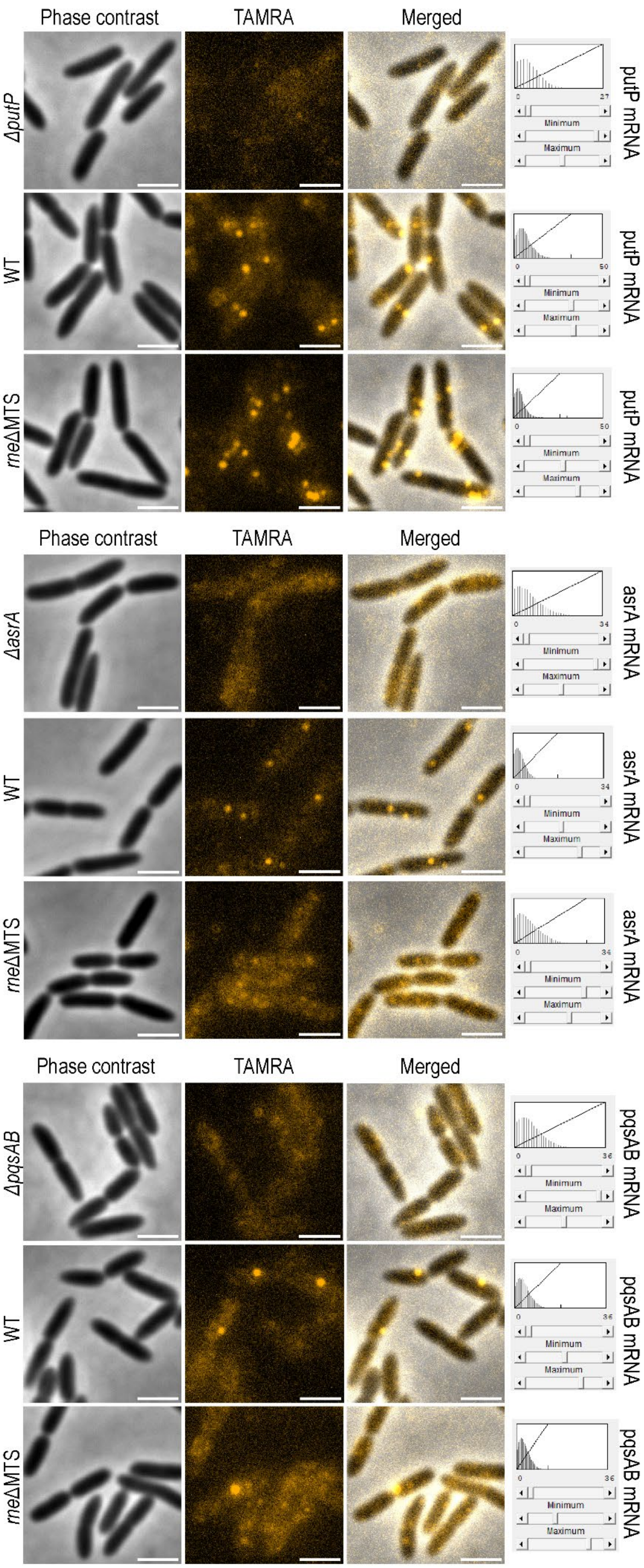

Figure S9: Visualisation of FISH signal by epifluorescence microscopy.
The phase contrast channel, fluorescence channel, or merged images, are shown separately. For each acquisition, the strain background is indicated on the left and the mRNA visualised is indicated on the right. Scale is 2 µm. The display range, adjusted in Fiji for each fluorescence image, is shown for reference.

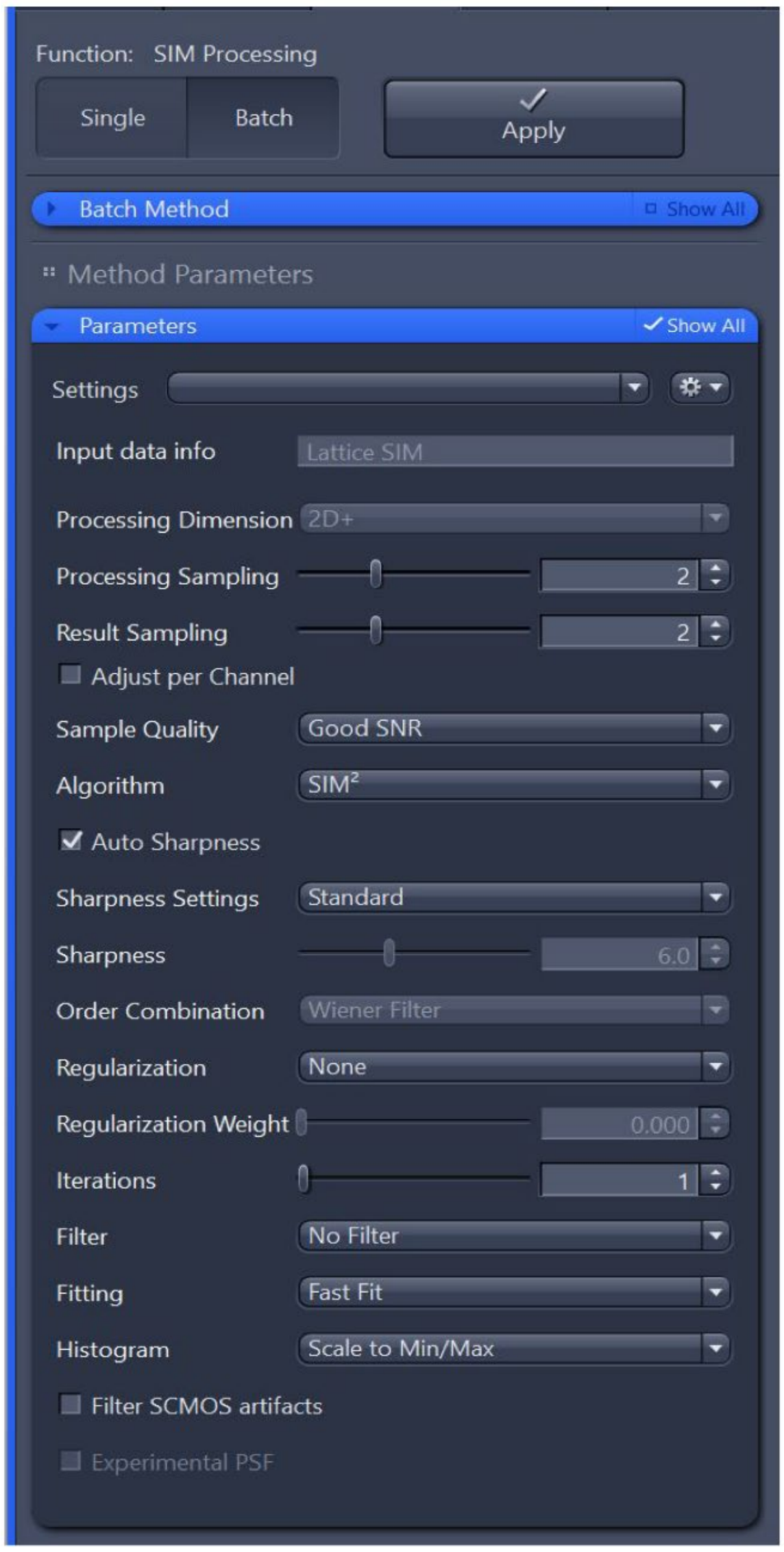

Figure S10: Reconstruction parameters used for SIM images in this study. The Zen Blue software was used for image acquisition and processing.

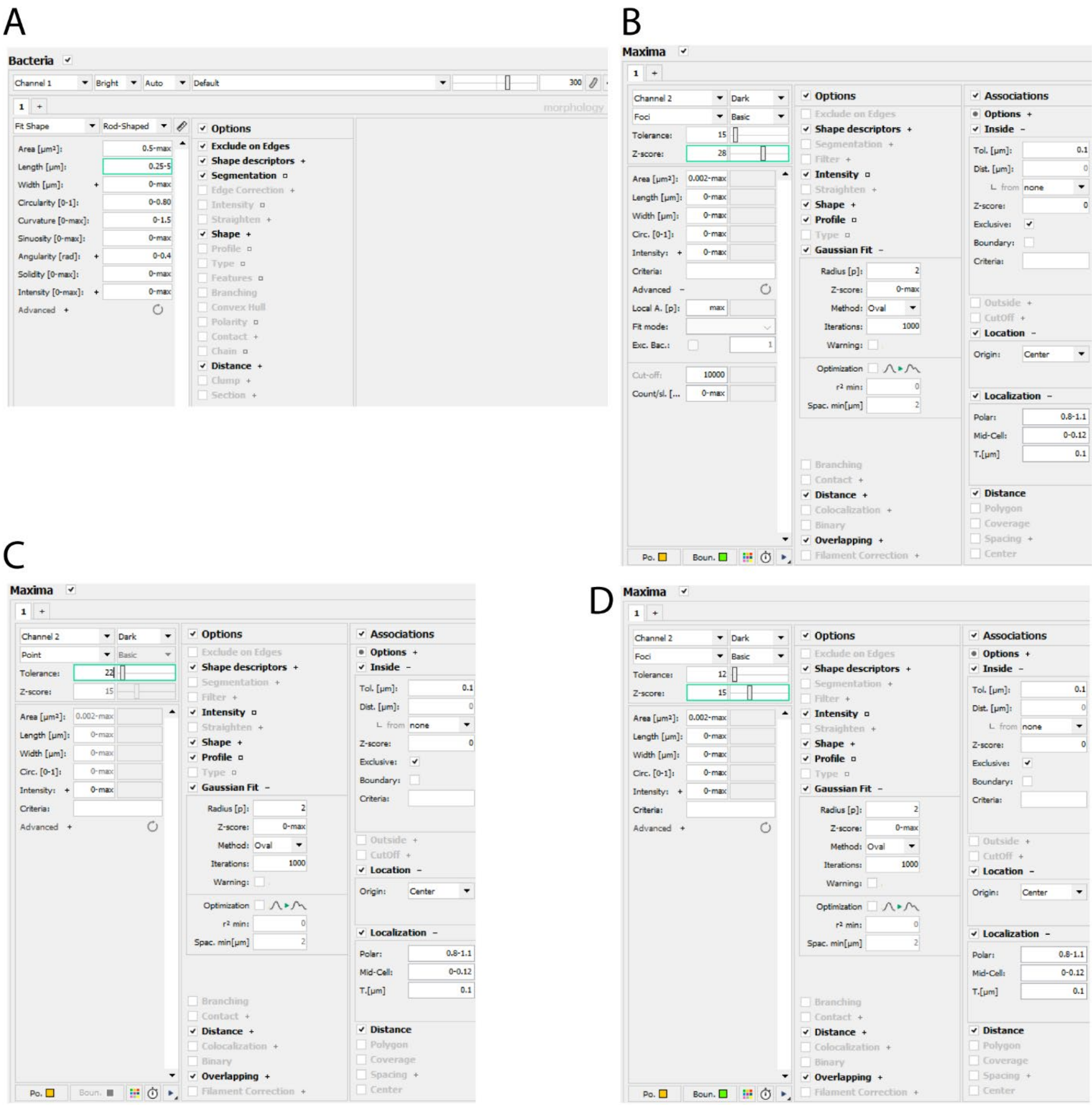

Figure S11: MicrobeJ detection parameters used in this study. Parameters that were manually adjusted between different images are highlighted by a green box. **(A)** Settings used for bacteria detection in all analysis done. The parameter of cell length (green box) was sometimes decreased to a max value of 4.5 or increased to a max value of 5.5 to ensure good cell detection in each image. When needed, additional manual segmentation was done as described in Materials and Methods. **(B)** Settings used for maxima detection when analysing msfGFP-tagged RNase E variants. A foci detection mode was chosen (using Z-score) to allow characterisation of foci morphology and subcellular localisation. The tolerance value was set to 15 for all image analysis but the Z-score was adjusted for each image to ensure optimal detection of foci outlines based on visual inspection. Z-score values set ranged from 26 to 58. **(C)** Settings used for maxima detection to obtain foci counts and intensities of mRNAs visualised by FISH. A point detection mode was chosen (no Z-score) to simplify the analysis and avoid manual input. The tolerance value was set identically for all images analysed for a given mRNA. For putP, asrA, and pqsAB, the tolerance values were 20, 22, and 28, respectively. **(D)** Settings used for maxima detection to plot subcellular localisation of mRNAs visualised by FISH. A foci detection mode was chosen (using Z-score) to ensure very low rate of false positives (at the cost of more false negatives) and very accurate detection of foci centre positioning thanks to detected foci outlines. The tolerance value was set to 12 for all image analysis but the Z-score was adjusted for each image to ensure optimal detection of foci outlines based on visual inspection. Z-score values set ranged from 7 to 15.

Table S1: Primers used in this study.

Primer code	Primer sequence (5' -> 3')
p199	GAATTCTATGCTCCTATATTG
p200	AAGCTTTTGCCATTCTC
p399	CAATATAGGAGCATAGAATTCTCCAGGTCGGTCGCATCTC
p400	CTTGCTGGTTTCAGCAGGCTTCGGCTCGGGCATCGGCTT
p401	AAGCCGATGCCCGAGCCGAAGCCTGCTGAAACCAGCAAG
p402	GAGAATGGCAAAAGCTTTCGCCATCGGTATCGTCCTG
p537	GGATCCTCTAGAGTCGACCTGC
p538	GAATTCGGTACCTTAATTA
p801	GTCGACTCTAGAGGATCCGACATCAAGATCAACGGTATCACCGAAG
p802	GAACCACCACCCACGCCGGAAGCTTCGG
p803	CGTGGGTGGTGGTTCCGTGTCTAAGGGCGA
p804	ACGACTCGACTTAATTAAGCTTGTGCCCCAGTTTGCT
p805	GCTTAATTAAGTCGAGTCGTGATCGATGAAAACAG
p806	GAAATTAATTAAGGTACCGAATTCGGCTTTCACGCCCTTGATGC
p971	CAGGTCGACTCTAGAGGATCCCAGGTCGGTCGCATCT
p972	AGGCTTTCCCGGGACGGCTCGGGCATCGGCTT
p973	TGCCCGAGCCGTCCCGGGAAAGCCTGGC
p974	TTTCAGCAGGCTTCTCGAGCCGCAGGTCG
p975	CCTGCGGCTCGAGAAGCCTGCTGAAACCAGCAAGC
p976	TTAATTAAGGTACCGAATTCTCGCCATCGGTATCGTCCTG
p977	GTTTCAACTTATTCGGCTCGGGCATCGGCTT
p978	ATGCCCGAGCCGAATAAGTTGAAACTTTTAGATGA
p979	TTTCAGCAGGCTTGTTTTGGTGCTTAACATATT
p980	TAAGCACCAAAACAAGCCTGCTGAAACCAGCAAGC
p1007	TCCTCCGAACAAGCGTTTGAGGAAGCCTTTCTTCGGCTCGGGCATCGGCTTG
p1008	AAGAAAGGCTTCCTCAAACGCTTGTTCGGAGGAAAGCCTGCTGAAACCAGCAAGC
p1037	CAGGTCGACTCTAGAGGATCCCGACGAGCTGAAGAAAGAGAACTTC
p1038	CGCCCGCAGGCGGTAGCGGGCCCTTGGTTG
p1039	AGGGCCCGCTACCGCCTGCGGGCGGGTAAAG
p1040	GGAAATTAATTAAGGTACCGAATTCCTCTGCCTGTTCGACGAATGC
p1074	CAGGTCGACTCTAGAGGATCCAGGTTCCGCACGATATGC
p1075	ACCTTGGCCACGTCGCTCATGAGGCACCT
p1076	CCTCATGAGCGACGTGGCCAAGGTGCTGT
p1077	GGAAATTAATTAAGGTACCGAATTCCGCGCAGGAAAAGCTTTC
p1078	CAGGTCGACTCTAGAGGATCCAGGTGTCCTCTTCGGCAG
p1079	TCGAAGCTGATCAGGGACATGACAGAACGTTCCC
p1080	TTCTGTCATGTCCCTGATCAGCTTCGATCCGTTC
p1081	GGAAATTAATTAAGGTACCGAATTCGCACACCACCAGCACTTTCTC
q65	CGTCGCGGCTACAAGTTCTCCA
q66	GGCTCGCGACCCATTTCCTG
q112	GATCCCGATACCGCCGTTTATCACTATC
q113	GCGGGACTTGGGATTGATCACG

q114	GCTTCAAGGTGATGGAAGGCCAAC
q115	GCCAGGGTGTAGGTCTTCCAGTC
q116	CTCCACCAACAACTTCTCCGACTACATC
q117	TTGCCGTTGTGCTCGGTCTG
q118	GCTGGGCTGCGAAGAAGCA
q119	CGTCCTGGCCTTCCTCGTACA
q120	GGATAGTCCACCTCCACCATGAACG
q121	TTCATGGACAATCCTCCGGAAGACC

Table S2: Plasmids used in this study.

**Name**	**Used for:**	**Insert(s) amplified with:**	**Template used in PCR**
pME3087, from [9]	Insert cloning	Linearised with p199-p200	Circular pME3087
pEXG2, from [10]	Insert cloning	Linearised with p537-p538	Circular pEXG2
pME3087_RNase E$^{\Delta MTS}$	Construction of PAO1 *rneΔMTS; rneΔMTS::2xStrep*; *rneΔMTS::msfGFP*; *rhl::msfGFP/ rneΔMTS::mCherry /pnp::mTagBFP2*	p399-p400; p401-p402.	PAO1 gDNA
pEXG2_RNase E$^{MinD\alpha}$	Construction of PAO1 *rneMinDα; rneMinDα::2xStrep*; *rneMinDα::msfGFP; rhl::msfGFP/ rneMinDα::mCherry /pnp::mTagBFP2*	p971-p1007; p1008-p976.	PAO1 gDNA
pEXG2_RNase E$^{PelC\alpha}$	Construction of PAO1 *rnePelCα::msfGFP*	p971-p972; p973-p974; p975-p976.	PAO1 gDNA
pEXG2_RNase E$^{FtsZ\alpha}$	Construction of PAO1 *rneFtsZα::msfGFP*	p971-p977; p978-p979; p976-p980.	PAO1 gDNA; *M. genitalium* FtsZ-containing plasmid
pEXG2_PNPase-mTagBFP2	Construction of PAO1 *rhl::msfGFP/ rne::mCherry /pnp::mTagBFP2*	p801-p802; p803-p804; p805-p806.	*pnp*-containing plasmid; mTagBFP2-containing plasmid
pEXG2_ΔPutP	Construction of PAO1 *ΔputP*	p1037-p1038; p1039-p1040.	PAO1 gDNA
pEXG2_ΔAsrA	Construction of PAO1 *ΔasrA*	p1074-p1075; p1076-p1077.	PAO1 gDNA
pEXG2_ΔPqsAΔPqsB	Construction of PAO1 *ΔpqsAB*	p1078-p1079; p1080-p1081.	PAO1 gDNA

Table S3: Oligos and primers used for preparation of the GC-EMOTE library.

Name	Sequence (5' -> 3')	Oligo type	Ordered from	Purification
BioVm1	Biotin-CUUCACCACACCCUAUCACHHHHHHHHHHHHHHHHHACAUA	RNA	IDT	Standard Desalting
DROCC_i7	CTGGAGTTCAGACGTGTGCTCTTCCGATCTNNNNNNNNNNCC	ssDNA	IDT	RNase-Free HPLC
pVmA	CTCTTTCCCTACACGACGCTCTTCCGATCTNTACACTTCACCACACCCTATCAC	ssDNA	Microsynth	IEX-HPLC
pVmB	CTCTTTCCCTACACGACGCTCTTCCGATCTNGTATCTTCACCACACCCTATCAC	ssDNA	Microsynth	IEX-HPLC
pVmC	CTCTTTCCCTACACGACGCTCTTCCGATCTNCGTCCTTCACCACACCCTATCAC	ssDNA	Microsynth	IEX-HPLC
pVmD	CTCTTTCCCTACACGACGCTCTTCCGATCTNAAGTCTTCACCACACCCTATCAC	ssDNA	Microsynth	IEX-HPLC
pVmE	CTCTTTCCCTACACGACGCTCTTCCGATCTNACACCTTCACCACACCCTATCAC	ssDNA	Microsynth	IEX-HPLC
pVmF	CTCTTTCCCTACACGACGCTCTTCCGATCTNGGTACTTCACCACACCCTATCAC	ssDNA	Microsynth	IEX-HPLC
pVmG	CTCTTTCCCTACACGACGCTCTTCCGATCTNTCGGCTTCACCACACCCTATCAC	ssDNA	Microsynth	IEX-HPLC
pVmH	CTCTTTCCCTACACGACGCTCTTCCGATCTNCAAGCTTCACCACACCCTATCAC	ssDNA	Microsynth	IEX-HPLC
pVmI	CTCTTTCCCTACACGACGCTCTTCCGATCTNTTGACTTCACCACACCCTATCAC	ssDNA	Microsynth	IEX-HPLC
pVmJ	CTCTTTCCCTACACGACGCTCTTCCGATCTNGCTGCTTCACCACACCCTATCAC	ssDNA	Microsynth	IEX-HPLC
pVmK	CTCTTTCCCTACACGACGCTCTTCCGATCTNCCGACTTCACCACACCCTATCAC	ssDNA	Microsynth	IEX-HPLC
pVmL	CTCTTTCCCTACACGACGCTCTTCCGATCTNCTCGCTTCACCACACCCTATCAC	ssDNA	Microsynth	IEX-HPLC
E1	AATGATACGGCGACCACCGAGATCTACACTCTTTCCCTACACGACG	ssDNA	Microsynth	IEX-HPLC
E2	CAAGCAGAAGACGGCATACGAGATCGTGATGTGACTGGAGTTCAGACGTGTGC	ssDNA	Microsynth	IEX-HPLC

Table S4: List of TAMRA-labelled FISH probes against putP, pqsAB, or asrA. Tm values were calculated using PerlPrimer[11].

FISH probe	FISH probe sequence	Length	GC	Predicted
putP_probe_1	GCGCCGTGACGAAGCT	16-mer	68%	63.44°C
putP_probe_2	TGGCCGAAGTAGCCCAG	17-mer	64%	61.55°C
putP_probe_3	CAGCCGCTCATATCGGAG	18-mer	61%	60.18°C
putP_probe_4	CGCGGTCTCGTAGGAGAG	18-mer	66%	61.72°C
putP_probe_5	AGGTCATGGAGATGCGGC	18-mer	61%	62.34°C
putP_probe_6	GATGCCGAAGAAGCCCAC	18-mer	61%	61.28°C
putP_probe_7	GGATGCGCCTGGAAGTAG	18-mer	61%	60.34°C
putP_probe_8	GAGGATGGCGGACAGCAA	18-mer	61%	62.57°C
putP_probe_9	CGAGGAGCAGACCAGCAG	18-mer	66%	62.60°C
putP_probe_10	CGGTTTTCCGGGTTGGAG	18-mer	61%	61.13°C
putP_probe_11	CAGGCATAGGACACCAGG	18-mer	61%	59.54°C
putP_probe_12	CGTTGTGCTCGGTCTGCA	18-mer	61%	63.42°C
putP_probe_13	GGTGAAGTAGTCGGGCAG	18-mer	61%	59.87°C
putP_probe_14	GGCTGTTGTCCTCGAAGC	18-mer	61%	61.19°C
putP_probe_15	GGTGTCGGTCCAGCTCAC	18-mer	66%	62.84°C
putP_probe_16	CGAGCATCACGATCACCG	18-mer	61%	61.10°C
putP_probe_17	CTCGGGGTTCTCGCTGAC	18-mer	66%	62.59°C
putP_probe_18	CAGCCAGATGGCGATCAC	18-mer	61%	61.04°C
putP_probe_19	ACTCCTGCTCCGCATCGT	18-mer	61%	63.92°C
putP_probe_20	TCCACAGGATCACCGTCG	18-mer	61%	61.53°C
putP_probe_21	GGTCCAGCCCAACAGGTT	18-mer	61%	62.38°C
putP_probe_22	TTGCGTGTGCAAGCAACG	18-mer	55%	63.00°C
putP_probe_23	GATCCAGCTTTCGGACAGG	19-mer	57%	60.62°C
putP_probe_24	TGTTGGTGGAGCGATATGC	19-mer	52%	60.62°C
putP_probe_25	ACCGAGGATGTAGTCGGAG	19-mer	57%	60.62°C
putP_probe_26	GGAAGCGCAGTAGATGGTG	19-mer	57%	60.99°C
putP_probe_27	CACCGAGGCAGAGGATCAT	19-mer	57%	61.62°C
putP_probe_28	CGATCCACGGGTTGAACAG	19-mer	57%	61.20°C
putP_probe_29	AGCTCAGGGTGCTCATGAC	19-mer	57%	62.15°C
putP_probe_30	ACAGCAGGGAGAACAGCAC	19-mer	57%	62.67°C
putP_probe_31	GGGGATGATCTCGTACAGG	19-mer	57%	59.11°C
putP_probe_32	CGAAGGTGCTCTCGAACAG	19-mer	57%	60.90°C
putP_probe_33	GAACACCAGGATCACCAGG	19-mer	57%	60.17°C
putP_probe_34	CCCAGACCAGTTCCAGCTG	19-mer	63%	62.74°C
putP_probe_35	AGGCTGAAGACGACGATGG	19-mer	57%	62.19°C
putP_probe_36	TCGATCAGCGCTGGGTTAC	19-mer	57%	62.55°C
putP_probe_37	CCTTGTAGAAGTCCTCGGTG	20-mer	55%	59.94°C
putP_probe_38	GTGGGCGTATTGACACTCAT	20-mer	50%	60.59°C
putP_probe_39	GCGATGTAGATCACGAAGGT	20-mer	50%	60.09°C
putP_probe_40	CGAACAGCCAGTTGAGGTAG	20-mer	55%	60.87°C
putP_probe_41	CGATGAAGGTGTAGGCGATG	20-mer	55%	61.07°C
putP_probe_42	ATCAGCGCGAAGATCATCAG	20-mer	50%	60.79°C
putP_probe_43	CTTGAGCATGTCGAAGCTGG	20-mer	55%	61.84°C
putP_probe_44	GAGATCACGCCGATGAAGGA	20-mer	55%	62.22°C
putP_probe_45	GATCGATTTCACCGAGTCGG	20-mer	55%	61.06°C
putP_probe_46	CTTGAGCATGCTCGTCGAAG	20-mer	55%	61.89°C
putP_probe_47	GGACTCCCCTATTGCTTGTTT	21-mer	47%	60.50°C
asrA_probe_1	TTCGGGATTAACGTCCTGGT	20-mer	50%	61.40°C
asrA_probe_2	GGGAAGAACGGGCGGTTG	18-mer	66%	63.85°C
asrA_probe_3	CACCAGTTCGAGGGTTTCC	19-mer	57%	60.77°C

asrA_probe_4	AGCTCCTTGATCGCGTTGA	19-mer	52%	62.12°C
asrA_probe_5	GAAGTCGGTGAGTGGCGA	18-mer	61%	62.14°C
asrA_probe_6	CGCTCTCGAGGATCGACA	18-mer	61%	61.35°C
asrA_probe_7	TGATGCTTGTCGAGGACCTT	20-mer	50%	61.69°C
asrA_probe_8	ATGCGATCCTTGATGTCGTC	20-mer	50%	60.73°C
asrA_probe_9	GCCTTTTCCAACTGCTTGG	19-mer	52%	60.10°C
asrA_probe_10	AGGGTTCTTCGTTGACGATC	20-mer	50%	60.23°C
asrA_probe_11	CGAATGGTGGTCGGTCTTG	19-mer	57%	61.20°C
asrA_probe_12	CAGGCTGTTGACGTCGAAG	19-mer	57%	61.48°C
asrA_probe_13	GTCGTTGGGACTGAAGCG	18-mer	61%	61.18°C
asrA_probe_14	CTCTTCGTCGATACGCTTGC	20-mer	55%	61.67°C
asrA_probe_15	GATGGATTTGCCGATGCTG	19-mer	52%	59.80°C
asrA_probe_16	GATCGAGTCGAGGGTGTTG	19-mer	57%	60.32°C
asrA_probe_17	GCAGTTTGACCACCGACTTG	20-mer	55%	62.26°C
asrA_probe_18	TGAGCTTGAAGCCACGGTT	19-mer	52%	62.60°C
asrA_probe_19	GGATTCCTTCATCACGTCGC	20-mer	55%	61.63°C
asrA_probe_20	GCCATACTTCTTCAGGTGCGA	21-mer	52%	62.94°C
asrA_probe_21	CACGTGCAGGTGGACGAA	18-mer	61%	63.06°C
asrA_probe_22	GGATCACGTAGAGGGTGGTC	20-mer	60%	61.88°C
asrA_probe_23	CGGAGGATTGTCCATGAAGAA	21-mer	47%	60.29°C
asrA_probe_24	ATCGGGGTCTTCGGATAGTC	20-mer	55%	61.03°C
asrA_probe_25	CCTTCACTTCGTCACTGGGAT	21-mer	52%	62.29°C
asrA_probe_26	TCCTTGAGGAAGAATTCGCG	20-mer	50%	60.59°C
asrA_probe_27	TCCTGCTGGATGATCTTCAAC	21-mer	47%	60.29°C
asrA_probe_28	ATCTCGCCCTTGAAACTACC	20-mer	50%	59.94°C
asrA_probe_29	CGACGCTGAAACGGTAGAAC	20-mer	55%	61.81°C
asrA_probe_30	ATCACCTCGACTTCCTTCAAC	21-mer	47%	60.22°C
asrA_probe_31	GTAGTGGTCGAGGAATTCGAC	21-mer	52%	60.69°C
asrA_probe_32	CCAGTTTTTCCTCGGAGATGTAG	23-mer	47%	61.19°C
asrA_probe_33	CGAGCTGTTTCTCCAACTGG	20-mer	55%	61.43°C
asrA_probe_34	CGATCTTCACCTTCGACTCG	20-mer	55%	60.71°C
asrA_probe_35	GATGTAGCTATAGGCGATCTCG	22-mer	50%	60.20°C
asrA_probe_36	GGTCGAAGAAGGTCGGGT	18-mer	61%	61.46°C
asrA_probe_37	GATCAGCTCGAAGATCTTCTGC	22-mer	50%	61.36°C
asrA_probe_38	GAGATAGTCGGGCAACTCTTC	21-mer	52%	60.42°C
asrA_probe_39	TAGTTCTTCAGTTCCTCGCTGTAC	24-mer	45%	62.40°C
asrA_probe_40	CGGCAAGACCTTCTCCAT	18-mer	55%	59.46°C
asrA_probe_41	GCGGACAGTTCCTTCTGCA	19-mer	57%	63.01°C
asrA_probe_42	ACTTCCACTTCCTTGCGCA	19-mer	52%	62.60°C
asrA_probe_43	GCTCTTGTCGTCCTTGGTGA	20-mer	55%	62.59°C
asrA_probe_44	AAATGCACGGTCAGCCCTT	19-mer	52%	63.00°C
asrA_probe_45	GCTTGTCCTTGCCGTACAC	19-mer	57%	61.79°C
asrA_probe_46	ATCACTTCCATCCGGTCGA	19-mer	52%	60.86°C
asrA_probe_47	GTCCTCGAGGTCCCTGG	17-mer	70%	60.87°C
asrA_probe_48	GCCTTTCTTCGGCACCTG	18-mer	61%	61.51°C
pqsAB_probe_1	GCGGAACAGAACCTCGGT	18-mer	61%	62.46°C
pqsAB_probe_2	CTCAGCGACGGTGCATC	17-mer	64%	61.00°C
pqsAB_probe_3	CAGCGAGGCATAGATGGC	18-mer	61%	60.81°C
pqsAB_probe_4	GCTGCTCAACAGCTCCCT	18-mer	61%	62.50°C
pqsAB_probe_5	CGGCCCAGAATTCGAACTC	19-mer	57%	61.28°C
pqsAB_probe_6	CACCAACAGCACGCCTTG	18-mer	61%	62.42°C
pqsAB_probe_7	GCCAGTAACCCGGACTCAG	19-mer	63%	62.49°C
pqsAB_probe_8	GTACCAGCCACCTGCGAA	18-mer	61%	62.48°C

pqsAB_probe_9	CGGCAGATGACGGCAGAT	18-mer	61%	62.69°C
pqsAB_probe_10	GTTGGCCAATGTGGACATGA	20-mer	50%	61.39°C
pqsAB_probe_11	GCGGTATCGGGATCGAAATC	20-mer	55%	61.21°C
pqsAB_probe_12	GTCTGGCCCCGATAGTGATAA	21-mer	52%	61.61°C
pqsAB_probe_13	GAAGGCGAGTCGTTCAACG	19-mer	57%	61.83°C
pqsAB_probe_14	GGGACTTGGGATTGATCACG	20-mer	55%	60.95°C
pqsAB_probe_15	GGCAGTCGGCAGCGATAT	18-mer	61%	62.76°C
pqsAB_probe_16	GACCAGGTTCTCCAGAACCC	20-mer	60%	62.34°C
pqsAB_probe_17	CACTCATAGCCAGGCAACG	19-mer	57%	61.28°C
pqsAB_probe_18	CACAGTGACGGTAGGCACC	19-mer	63%	63.06°C
pqsAB_probe_19	CAGCACATGCAATTGGCTG	19-mer	52%	60.91°C
pqsAB_probe_20	TGGGAGAGAATGTAGGTCCG	20-mer	55%	60.89°C
pqsAB_probe_21	ATGGAAGGCACTCCAATCGA	20-mer	50%	61.77°C
pqsAB_probe_22	GAGGTGTATTGCAGGAAACAGG	22-mer	50%	61.71°C
pqsAB_probe_23	GCAGAAACCGAGCGTGTTG	19-mer	57%	62.70°C
pqsAB_probe_24	AGCAACTCCGTAGCGAACG	19-mer	57%	63.09°C
pqsAB_probe_25	AATCGAATACAGCCGGTCTC	20-mer	50%	60.09°C
pqsAB_probe_26	TGCCATAGCCGAAGAACATCT	21-mer	47%	62.09°C
pqsAB_probe_27	ACCAGGGAAAGAACAGGCTG	20-mer	55%	62.57°C
pqsAB_probe_28	TTCCTCGATAGTGTGTCCTTCG	22-mer	50%	62.03°C
pqsAB_probe_29	GGCCATTCACCTTGAACAGATC	22-mer	50%	62.03°C
pqsAB_probe_30	GGCAGGTAGGAACCAGAACC	20-mer	60%	62.69°C
pqsAB_probe_31	AACAGGGTCGGACGCAAG	18-mer	61%	62.78°C
pqsAB_probe_32	CATGTGCGAGGGAATCTGTT	20-mer	50%	60.88°C
pqsAB_probe_33	AAGGTTGTCGTGATAAAGGGTG	22-mer	45%	61.06°C
pqsAB_probe_34	GAAGACATGCGCGCGATTC	19-mer	57%	62.95°C
pqsAB_probe_35	CAGGCTCGAGTCGGTGAG	18-mer	66%	62.27°C
pqsAB_probe_36	GACCAGGACGTTGCGATAG	19-mer	57%	60.69°C
pqsAB_probe_37	TTGAGGTGTCCCTTGACGT	19-mer	52%	61.25°C
pqsAB_probe_38	CTTCGGGCAATCCAGGTTG	19-mer	57%	61.52°C
pqsAB_probe_39	CCAGACGGGTGAATCCGG	18-mer	66%	62.62°C
pqsAB_probe_40	CTCCAGACACACATAGGATGG	21-mer	52%	60.08°C
pqsAB_probe_41	ATCAGATGGTCGGGAGACAG	20-mer	55%	61.25°C
pqsAB_probe_42	AGGGTATCGACCACGTAGAG	20-mer	55%	60.66°C
pqsAB_probe_43	GCGAATATCGGTATTCAGCG	20-mer	50%	59.47°C
pqsAB_probe_44	CAGTTCAGTTGCGGGAAGTC	20-mer	55%	61.71°C
pqsAB_probe_45	GCGAGAAATCGTCGAGCAAAG	21-mer	52%	62.70°C
pqsAB_probe_46	AGCAGTTCATCCAGACGGT	19-mer	52%	61.40°C
pqsAB_probe_47	AGGATCTGGTTGTCGTCCA	19-mer	52%	60.71°C
pqsAB_probe_48	GAATCAACATGCCCGTTCCT	20-mer	50%	61.17°C

References

1. Szeto, T.H., Rowland, S.L., Habrukowich, C.L., and King, G.F. (2003). The MinD Membrane Targeting Sequence Is a Transplantable Lipid-binding Helix*. Journal of Biological Chemistry *278*, 40050-40056. https://doi.org/10.1074/jbc.M306876200.
2. Zhou, H., and Lutkenhaus, J. (2003). Membrane Binding by MinD Involves Insertion of Hydrophobic Residues within the C-Terminal Amphipathic Helix into the Bilayer. Journal of Bacteriology *185*, 4326-4335. 10.1128/jb.185.15.4326-4335.2003.
3. Kowalska, K., Soscia, C., Combe, H., Vasseur, P., Voulhoux, R., and Filloux, A. (2010). The C-terminal amphipathic α-helix of Pseudomonas aeruginosa PelC outer membrane protein is required for its function. Biochimie *92*, 33-40. https://doi.org/10.1016/j.biochi.2009.10.004.
4. Dutta, S., Poddar, S., Chakraborty, J., Srinivasan, R., and Gayathri, P. (2025). Membrane Binding and Cholesterol Sensing Motif in Mycoplasma genitalium FtsZ: A Novel Mode of Membrane Recruitment for Bacterial FtsZ. Biochemistry *64*, 1864-1877. 10.1021/acs.biochem.4c00543.
5. Aït-Bara, S., Carpousis, A.J., and Quentin, Y. (2015). RNase E in the γ-Proteobacteria: conservation of intrinsically disordered noncatalytic region and molecular evolution of microdomains. Mol Genet Genomics *290*, 847-862. 10.1007/s00438-014-0959-5.
6. Gautier, R., Douguet, D., Antonny, B., and Drin, G. (2008). HELIQUEST: a web server to screen sequences with specific alpha-helical properties. Bioinformatics *24*, 2101-2102. 10.1093/bioinformatics/btn392.
7. Chao, Y., Li, L., Girodat, D., Förstner, K.U., Said, N., Corcoran, C., Śmiga, M., Papenfort, K., Reinhardt, R., Wieden, H.-J., et al. (2017). In Vivo Cleavage Map Illuminates the Central Role of RNase E in Coding and Non-coding RNA Pathways. Molecular Cell *65*, 39-51. https://doi.org/10.1016/j.molcel.2016.11.002.
8. Redder, P. (2018). Chapter Fifteen - Mapping 5′-Ends and Their Phosphorylation State With EMOTE, TSS-EMOTE, and nEMOTE. In Methods in Enzymology, A.J. Carpousis, ed. (Academic Press), pp. 361-391. https://doi.org/10.1016/bs.mie.2018.08.011.
9. Ye, R.W., Haas, D., Ka, J.O., Krishnapillai, V., Zimmermann, A., Baird, C., and Tiedje, J.M. (1995). Anaerobic activation of the entire denitrification pathway in Pseudomonas aeruginosa requires Anr, an analog of Fnr. Journal of Bacteriology *177*, 3606-3609. 10.1128/jb.177.12.3606-3609.1995.
10. Rietsch, A., Vallet-Gely, I., Dove, S.L., and Mekalanos, J.J. (2005). ExsE, a secreted regulator of type III secretion genes in Pseudomonas aeruginosa. Proceedings of the National Academy of Sciences *102*, 8006-8011. 10.1073/pnas.0503005102.
11. Marshall, O.J. (2004). PerlPrimer: cross-platform, graphical primer design for standard, bisulphite and real-time PCR. Bioinformatics *20*, 2471-2472. 10.1093/bioinformatics/bth254.
